# Extracellular Vesicles Play a Central Role in Cerebral Venous Disease‐Associated Brain Atrophy

**DOI:** 10.1002/advs.202301574

**Published:** 2023-07-12

**Authors:** Jia‐Yu Wang, Jing‐Ying Li, Dan Luo, Mei‐Ying Huang, Dong‐Hui Ao, Xin‐nan Liu, Xia Wang, Wei Ge, Yi‐Cheng Zhu

**Affiliations:** ^1^ Department of Neurology State Key Laboratory of Complex Severe and Rare Diseases Peking Union Medical College Hospital Chinese Academy of Medical Sciences and Peking Union Medical College Beijing 100730 China; ^2^ Department of Immunology Institute of Basic Medical Sciences Chinese Academy of Medical Sciences School of Basic Medicine Peking Union Medical College Beijing 100005 China; ^3^ Department of Histology and Embryology Basic Medical College China Medical University Shenyang 110122 China

**Keywords:** brain atrophy, cerebral venous disease, deep medullary veins, extracellular vesicles, proteomics

## Abstract

Cerebral venous abnormalities, distinct from traditional arterial diseases, have been linked to brain atrophy in a previous community‐based cohort study, specifically in relation to the reduction of deep medullary veins (r‐DMVs). To better understand the properties and biological functions of serum extracellular vesicles (EVs) in cerebral venous disease‐associated brain atrophy, EVs are extracted from the serum of both participants with r‐DMV and normal controls and analyzed their proteomic profiles using Tandem Mass Tag label quantitation analysis. Phenotypic experiments showed that EVs from individuals with r‐DMVs are able to disrupt the normal functions of neurons, endothelial cells, and smooth muscle cells, and induce A1 reactive astrocytes. Additionally, this study provided a comprehensive characterization of the proteomic profile of DMV EVs and found that the collagen hydroxyproline is upregulated, while complement C3 is downregulated in the r‐DMV group, suggesting that r‐DMV may not be a simple pathological phenomenon and highlighting the potential involvement of EVs in the progression of brain atrophy in r‐DMVs which has implications for the development of future therapeutic strategies.

## Graphical abstract

Reduced deep medullary veins (r‐DMVs) are associated with brain atrophy. This research revealed extracellular vesicles (EVs) from r‐DMVs showed negative regulatory effects on various types of brain cells. Moreover, it presented the first comprehensive characterization of the proteomic profile of r‐DMV EVs, uncovering down‐regulated complement C3 in r‐DMV group. These findings indicate r‐DMVs may not merely be a simple pathological occurrence.

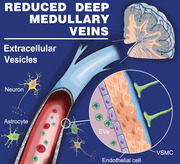

## 1 Introduction

Venous collagenosis (VC), caused by abnormal collagenous thickening of the venous walls, was first described in 1995 in a pathological study of postmortem brains and found to be most prominent in regions adjacent to the lateral ventricles.^[^ ^1^ ^]^ VC is classified as an etiopathogenic type of cerebral small vessel disease (CSVD). Recently, venules have attracted considerable research interest because of their involvement in perivenous lymphatic drainage in neurodegenerative diseases.^[^ ^2^ ^]^ VC has been found in some neurodegenerative diseases, such as Alzheimer's disease,^[^ ^3^ ^]^ and has been described as the pathogenesis of white matter hyperintensity seen in Magnetic resonance imaging (MRI).

VC may lead to venous stenosis and obstruction, accounting for the decreased number of venules visualized in susceptibility‐weighted imaging (SWI) in MRI. Consequently, an increasing number of in vivo studies have investigated cerebral venous diseases by counting visible venules, especially deep medullary veins (DMVs). DMVs are the most feasible to be counted because of their regular arrangement perpendicular to the ventricles. Previous studies have revealed that a decreased number of DMVs is independently related to white matter hyperintensity as well as brain atrophy.^[^ ^4^ ^]^ However, common vascular risk factors are mainly derived from arterial studies, leaving risk factors relative to VC largely unknown. We previously investigated DMVs using brain MRI in a general population and found a stable correlation between reduced DMV numbers and increased age, but no significant association between DMV numbers and any other common vascular risk factors.^[^ ^5^, ^6^ ^]^ These results highlight the importance of cerebral venous abnormalities and their differences from traditional arterial diseases and warrant further investigation of associated venous risk factors and biomarkers.

Extracellular vesicles (EVs) are small cargo‐bearing vesicles that are released by cells into the extracellular space. Intercellular communication through EVs appears to be involved in the pathogenesis of various disorders, including neurodegeneration and inflammatory diseases.^[^ ^7^ ^]^ EVs can serve as potential biomarkers because their content is a molecular signature of the cell of origin, and exosome‐based diagnostic tests are being used for the early detection of some diseases.^[^ ^8^ ^]^ Investigating serum EVs from patients with DMV‐reduction and brain atrophy may provide clues for potential risk factors related to venous pathology.

To explore this issue, we first conducted a proteomic analysis of serum EVs to identify potential biomarkers in a population‐based cohort of 1586 participants comprising participants with reduced DMV and an age‐ and sex‐matched control group. We then studied the biological properties of EVs and their physiological functions in DMV reduction and brain atrophy.

## 2 Results

### 2.1 Cerebral Deep Medullary Vein Reduction (r‐DMV) is Associated with Brain Atrophy

**Figure 1:**
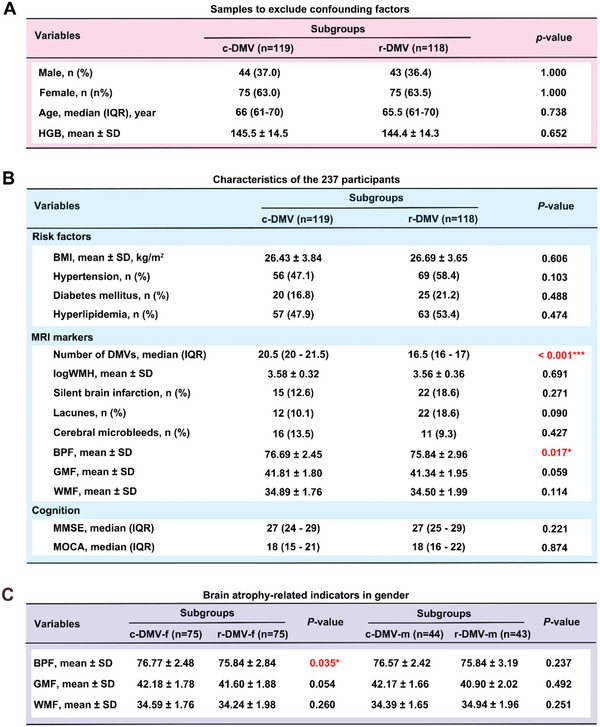
The reduction of deep medullary vein (r‐DMV) is associated with brain atrophy. A) Samples to exclude confounding factors. B) Characteristics of the 237 participants. C) Brain atrophy‐related indicators for each sex. HGB, hemoglobin; BMI, body mass index; DMV, deep medullary vein; WMH, white matter hyperintensity; BPF, brain parenchyma fraction; GMF, gray matter fraction; WMF, white matter fraction; MMSE, Mini‐Mental State Examination; MOCA, Montreal Cognitive Assessment. Binomial variables are expressed as n (%); continuous variables with normal distribution are expressed as mean ± standard deviation (SD); continuous variables without normal distribution are expressed as median (interquartile range, IQR); c‐DMV, control DMV; r‐DMV, reduced DMV. *P < 0.05, ***P < 0.001. Student's *t*‐test was used as appropriate.

To understand the relationship between DMV number and brain atrophy, we selected the 25% of the Shunyi cohort with the largest number of veins (DMV ≥20) as the control DMV group (c‐DMV), and the 25% population with the fewest veins (DMV ≤17.5) as the r‐DMV group. It was previously reported that the number of DMV decreases with age^[^ ^6^ ^]^; therefore, we matched the sex and age of the two groups to control for confounding factors. Finally, 119 participants were included in the c‐DMV group (37.0% males), and 118 subjects were included in the r‐DMV group (36.4% males). Because of the effect of deoxygenated hemoglobin on DMV susceptibility, we measured hemoglobin (HGB) levels to exclude the potential influence of deoxygenated hemoglobin on DMV imaging. As shown in **Figure** **1**A, there were no significant differences between the c‐DMV and r‐DMV groups after excluding the confounding factors.

We also identified indicators of traditional cerebrovascular disease risk, such as BMI, hypertension, diabetes mellitus, and hyperlipidemia. There were no significant differences in these indicators between the c‐DMV and r‐DMV groups, suggesting that DMV reduction and traditional cerebrovascular disease have no shared risk factors. We found that the BPF in the r‐DMV group was significantly lower than that in the c‐DMV group. In our previous cohort study, we detected a positive correlation between BPF and DMV numbers.^[^ ^5^ ^]^ Therefore, we performed mini‐mental state examination (MMSE) and montreal cognitive assessment (MOCA) cognitive analyses, which showed no significant differences in terms of cognitive function between the two groups (Figure 1B). We reanalyzed the included population by sex and found a greater decline in the Brain parenchymal fraction (BPF) in females (Figure 1C). The clinical characteristics of participants with c‐DMV (*n* = 119) and r‐DMV (*n* = 118) information are shown in Table S1, Supporting Information.

### 2.2 Extraction Efficiency and Verification of EVs

**Figure 2:**
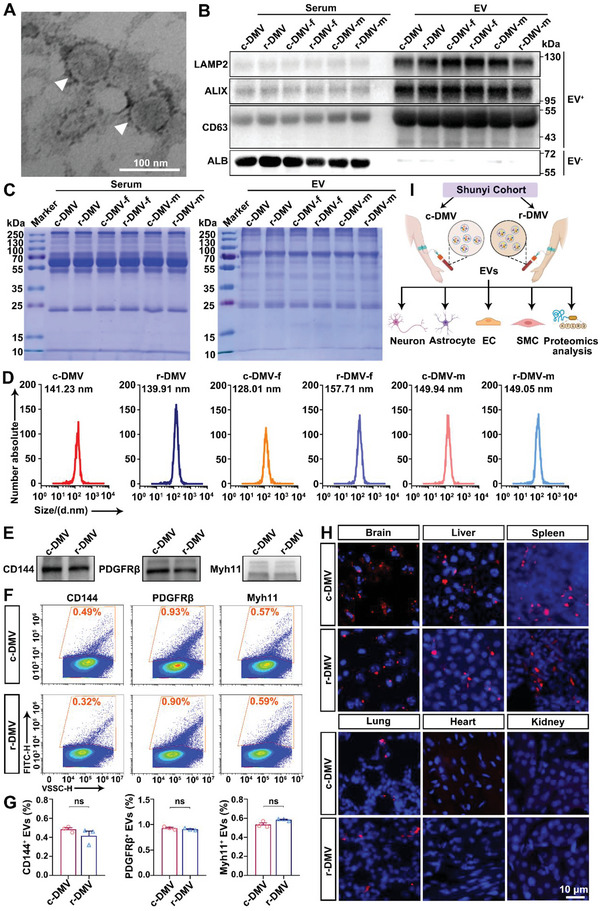
Extraction efficiency and verification of EVs. A) Electron microscope image showing the intact morphology of EVs. B) Western blot showing enrichment of the EV markers LAMP2, ALIX, CD63, and ALB (albumin). C) Coomassie Blue staining was used as a control to access standardized loading. D) Nanoparticle tracking analyses (NTA) of serum EVs. E) Western blot and F,G) flow cytometry analysis of endothelial‐specific marker CD144, pericyte‐specific marker PDGFRβ, and vascular smooth muscle cell marker Myh11 in serum EVs derived from c‐DMV and r‐DMV. F) Representative flow cytometry plots displaying CD144^+^, PDGFRβ^+^, and Myh11^+^ EVs. G) Quantification of cell‐specific EVs from images; *n* = 3 per group. H) EV‐positive organs and EV‐negative organs intravenous injection of fluorescence labeled EVs. Representative images of the brain, liver, spleen, lung, heart, and kidney after intravenous EV injection are shown. The nuclei of the cells are marked in blue (DAPI). EVs appear in red (PKH26). I) Schematic diagram of the experimental design. c‐DMV, control DMV; r‐DMV, reduced DMV; c‐DMV‐f, females from control DMV; r‐DMV‐f, females from reduced DMV group; c‐DMV‐m, males from control DMV group; and r‐DMV‐m, males from reduced DMV group. ns, not significant. Student's *t*‐test was used as appropriate. Data represent the mean ± SEM.

**Figure 3:**
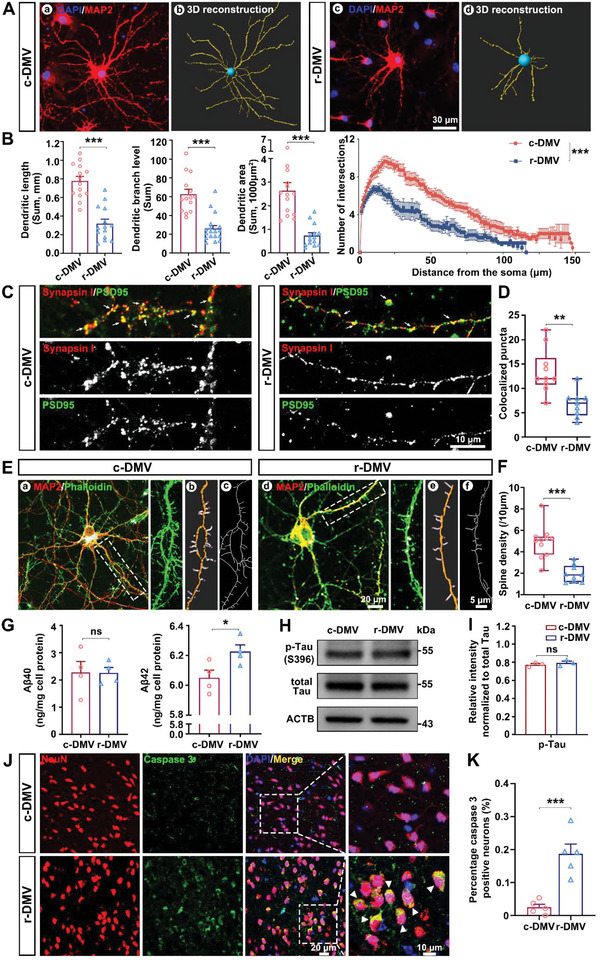
EVs of r‐DMV‐treated neurons lose many normal functions. A) Representative confocal images of rat primary neurons immunostained with neuron marker MAP2 (red) and DAPI (blue) (a,c). Imaris‐rendered neuron morphology is immunostained with MAP2 (yellow) in primary culture neurons (b,d). B) Dendrite length, branch level, and area, and Sholl analysis quantification in neurons; *n* = 14 neurons per group. C) Representative images of rat primary neurons immunostained with the pre‐ and post‐synaptic markers synapsin I (red) and PSD95 (green), respectively. Co‐localization of staining (yellow puncta) was counted as a structural synapse; example synapses are indicated with arrows. D) Quantification of synapse density from images; *n* = 9–10 dendrites. E) Representative confocal images of dendritic spines of rat primary neurons immunostained with phalloidin (green) (a,d). Images of the selected region of interest (ROI) were reconstructed using Imaris (b,e). Skeleton images of the selected ROI using Image J software (c,f). F) Quantification of spine density from images; *n* = 9–11 dendrites. Images in (A, C, and E) were adjusted for brightness and contrast. A–F) Rat primary neurons treated with serum extracellular vesicles derived from c‐DMV (pooled samples of 119 participants) or r‐DMV (pooled samples of 118 participants). G) SH‐SY5Y treated with EVs analyzed by ELISA using antibodies for the specific detection of Aβ40 and Aβ42. H) Western blot analysis confirmed no significant difference in p‐tau (S396) between serum EVs from c‐DMV and r‐DMV, with total tau as the internal control of protein expression. I) Densitometric analyses of the immunoreactivities to the antibodies shown in (H). G‐I) SH‐SY5Y treated with serum extracellular vesicles derived from c‐DMV (pooled samples of 119 participants) or r‐DMV (pooled samples of 118 participants). J) Representative confocal images of mouse neurons immunostained with neuron marker NeuN (red), cleaved caspase‐3 (green), and DAPI (blue). Co‐localization of staining (yellow) was counted as the apoptotic neurons; for example, apoptotic neurons are indicated with arrows. K) Quantification of apoptotic neurons from images; *n* = 5 per group. c‐DMV, control DMV; r‐DMV, reduced DMV. **P < 0.01, ***P < 0.001; ns, not significant. Student's *t*‐test was used as appropriate. Data represent the mean ± SEM.

Proteins in EVs have been associated with brain atrophy in patients with clinically manifested vascular disease,^[^ ^9^ ^]^ suggesting that EVs may be involved in the process of DMV reduction‐associated brain atrophy. We first isolated the EVs from pooled serum samples of the participants using an IZON qEV original column. Ultrastructural analysis by transmission electron microscopy showed the serum‐derived EVs were in the size range of 100–200 nm in diameter and displayed a lipid bilayer membrane with a cup‐shaped morphology (**Figure** **2**A). EV fractions were validated based on the enrichment of positive EV markers, including lysosomal‐associated membrane protein 2 (LAMP2), ALG‐2 interacting protein X (ALIX), and CD63.^[^ ^10^, ^11^, ^12^ ^]^ Contrastingly, the serum contained a high level of albumin (ALB), which was hardly detectable in the EV fractions and therefore, served as a negative EV marker (Figure 2B). Coomassie Blue staining of separated proteins confirmed that equal amounts of proteins were loaded from each sample (**Figure** **3**C). Nanoparticle tracking analysis (NTA) of the serum‐derived EVs showed median sizes in the control DMV (c‐DMV), reduced DMV (r‐DMV), females from control DMV group (c‐DMV‐f), females from reduced DMV group (r‐DMV‐f), males from control DMV group (c‐DMV‐m), and males from reduced DMV group (r‐DMV‐m) of 141.23 nm, 139.91 nm, 128.01 nm, 157.71 nm, 149.94 nm, and 149.15 nm, respectively (Figure 2D). The particle diameter ranged from 100 to 200 nm, which was consistent with the size of EVs. Overall, these data revealed that the purity of our extracted EVs conformed with the Minimal Information for Studies of Extracellular Vesicles 2018 (MISEV2018).^[^ ^13^ ^]^ We performed western blotting (WB) and flow cytometry assays of different cell‐specific markers, endothelial cells (CD144)^[^ ^14^ ^]^, pericytes (PDGFRβ)^[^ ^15^, ^16^ ^]^, and vascular smooth muscle cells (Myh11)^[^ ^17^ ^]^ to investigate the changes of EV types between c‐DMV and r‐DMV group. The results revealed no significant differences between c‐DMV and r‐DMV groups (c‐DMV vs. r‐DMV): CD144^+^ (0.483% ± 0.018% vs. 0.417% ± 0.048%), PDGFRβ^+^ (0.930% ± 0.017% vs. 0.907% ± 0.012%), and Myh11^+^ (0.533% ± 0.019% vs. 0.583% ± 0.007%) (Figure 2E‐G). In order to investigate whether EVs from r‐DMVs have an impact on brain cells, we first verified whether serum‐derived EVs from c‐DMVs and r‐DMVs can penetrate the brain, PKH26‐labelled EVs were intravenously injected into C57/BL6 mice, we found that serum‐derived EVs not only penetrated the brain but also entered organs such as the liver, spleen, and lungs (Figure 2H). The schematic diagram of the experimental design was shown in Figure 2I.

### 2.3 r‐DMV‐EVs‐Treated Neurons Lose Many Normal Functions

EVs absorbed by neurons have been reported to modulate neuronal functions.^[^ ^18^ ^]^ Since brain atrophy is caused by the loss of neurons and connections between neurons, we suspected that EVs from the r‐DMV population may affect the survival of neurons. To test this hypothesis, we isolated pooled serum‐derived EVs from r‐DMV participants and investigated their effects on neurons using c‐DMV EVs as controls. Neuronal morphology plays a significant role in determining neuronal function and communication.^[^ ^19^ ^]^ Thus, we first evaluated the morphological changes in rat cortical neurons treated with serum‐derived EVs using the neuron‐specific microtubule‐associated protein 2 (MAP2), which is an abundant protein of cytoskeletal component, to image the neurons^[^ ^20^ ^]^ (Figure 3A‐a,c). Imaris software was used to visualize the architecture of the neuron network and neurite outgrowth (Figure 3A‐b,d). We found that EVs of r‐DMV impaired the dendritic arborization of the neurons, which was reflected in a shorter total dendrite length, fewer dendrite branches, smaller dendrite areas, and reduced intersection numbers in the Sholl analysis (Figure 3B). We also detected the effect of EVs on synapse and dendritic spine formation by treating primary cortical neurons for 24 h with 10 µg r‐DMV or c‐DMV EVs and examining the co‐localization of presynaptic synapsin I and postsynaptic PSD 95. We observed a decrease in the density of colocalized synapsin‐PSD 95 puncta in neurons treated with r‐DMV EVs compared with those treated with the c‐DMV EVs (Figure 3C,D). We then performed phalloidin staining to explore the function of r‐DMV EVs in neuronal dendritic spines. Treatment of cortical neurons with r‐DMV EVs induced a decrease in spines compared with the effects of the c‐DMV EVs (Figure 3E,F). Deposits of fibrillar β‐amyloid peptide (Aβ) and intracellular bundles of self‐assembled hyperphosphorylated tau proteins (p‐tau) are two hallmark lesions of neurodegenerative diseases, such as Alzheimer's disease.^[^ ^21^ ^]^ We also investigated the association between r‐DMV EVs and neurodegeneration by analyzing the expression of Aβ42 and Aβ40 in treated SY5Y cells. Treatment with r‐DMV EVs significantly increased the expression level of Aβ42 in SY5Y cells compared with the effects of c‐DMV EVs treatment, while there was no significant difference in the expression level of Aβ40 between the two groups (Figure 3G). Furthermore, there were no significant differences in the protein levels of total tau and p‐tau (S396) between the two groups (Figure 3H,I). We further substantiate the impact of r‐DMV EVs on neurons in vivo by intravenous injection of EVs into mice. Our findings revealed an augmented expression of cleaved caspase 3, suggesting that r‐DMV EVs exhibited a greater propensity to provoke neuronal apoptosis in comparison to c‐DMV EVs (Figure 3J,K). Taken together, these findings suggest that r‐DMV EVs play a critical role in brain atrophy by disrupting normal neuronal function.

### 2.4 r‐DMV EVs Induce A1 Reactive Astrocytes

**Figure 4:**
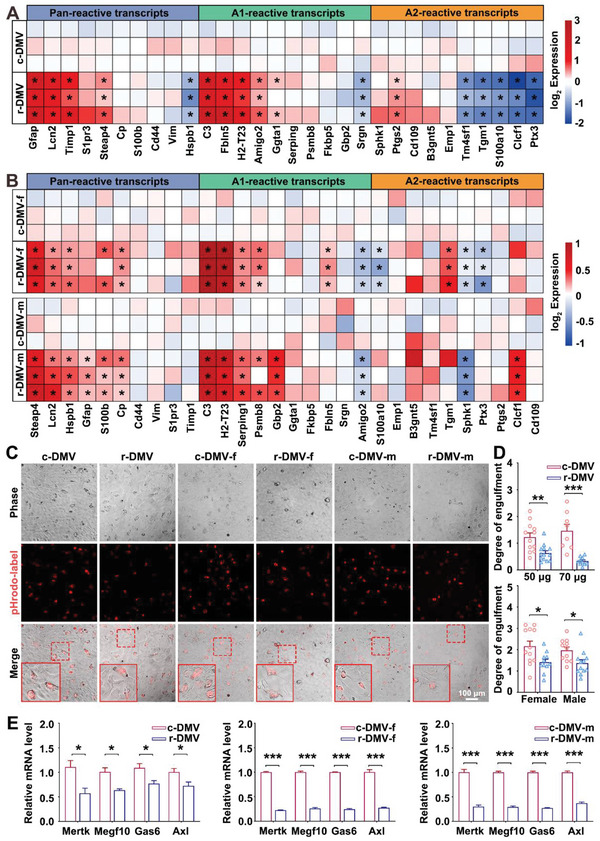
The r‐DMV EVs induced A1 astrocyte reactivity. A,B) Heatmap of pan‐reactive and A1‐ and A2‐ reactive transcripts in astrocytes treated with c‐DMV and r‐DMV EVs (A); and in astrocytes treated with c‐DMV‐f, r‐DMV‐f, c‐DMV‐m, and r‐DMV‐m EVs (B). C) Phase and fluorescent images of cultured astrocytes engulfing pHrodo‐conjugated synaptosomes among c‐DMV, r‐DMV, c‐DMV‐f, r‐DMV‐f, c‐DMV‐m, and r‐DMV‐m groups. D) Quantification of (C); *n* = 8–12. E) Quantitative PCR analysis of astrocyte‐specific phagocytic receptors (Mertk, Megf10, Gas6, and Axl) decreased in A1 reactive astrocytes. c‐DMV, control DMV; r‐DMV, reduced DMV; c‐DMV‐f, females from control DMV group; r‐DMV‐f, females from reduced DMV group; c‐DMV‐m, males from control DMV group; and r‐DMV‐m, males from reduced DMV group. *P < 0.05, **P < 0.01, ***P < 0.001; ns, not significant. Student's *t*‐test was used as appropriate. Data represent the mean ± SEM.

Astrocytes play supportive roles in central nervous system (CNS) functions, such as neurotransmitter homeostasis and synapse development.^[^ ^22^ ^]^ Some CNS diseases can induce two different reactive astrocyte states, A1 and A2.^[^ ^23^ ^]^ A1 reactive astrocytes amplify inflammatory responses and produce neurotoxicity effects, which is involved in brain atrophy.^[^ ^24^ ^]^ The purity of the primary cultured astrocytes isolated from the neonatal rat cortex was confirmed to be satisfactory for use in our study using GFAP staining (Figure S1A, Supporting Information). Next, we determined the effects of r‐DMV EVs on the reactive state of astrocytes by assessing mRNA levels of pan‐reactive, A1‐specific, and A2‐specific markers. The transcript level of pan‐reactive (Gfap, Lcn2, Timp1, S1pr3, and Steap4) and A1‐reactive (C3, Fbln5, H2‐T23, Amigo2, Ggta1, Serping, and Psmb8) were significantly increased, whereas those of A2‐reactive markers (Tm4sf1, Tgm1, S100a10, Clcf1, and Ptx3) were significantly decreased in r‐DMV EVs treated astrocytes (**Figure** **4**A and S1B, Supporting Information). Furthermore, both r‐DMV‐f and r‐DMV‐m EVs significantly increased the mRNA expression levels of pan‐reactive and A1‐specific markers (Figure 4B and S1C, Supporting Information). Astrocytes are not only important for the formation of synapses, but are also essential for the phagocytic elimination of synapses.^[^ ^25^ ^]^ It has been previously reported that A1 reactive astrocytes have decreased phagocytic capacity.^[^ ^23^ ^]^ Therefore, to compare the phagocytic abilities of astrocytes treated with r‐DMV‐EVs and c‐DMV‐EVs, we measured synaptosome engulfment. The r‐DMV‐EV‐treated astrocytes engulfed fewer synaptosomes than the c‐DMV‐EV‐treated astrocytes. Both r‐DMV‐f and r‐DMV‐m EVs treatments also significantly decreased the phagocytic ability of astrocytes compared with the effects of c‐DMV‐f and c‐DMV‐m EVs (Figure 4C,D). This phagocytic deficit corresponded to decreased mRNA expression level of phagocytic receptors including Mertk, Megf10, Gas6, and Axl (Figure 4E). These data reveal that r‐DMV EVs are capable of inducing A1 reactive astrocytes. Taken together, these data indicate that r‐DMV EVs may be involved in brain atrophy by inducing A1 reactive astrocytes.

### 2.5 r‐DMV‐derived EVs Induce Endothelial Cell and Smooth Muscle Cell Dysfunction

**Figure 5:**
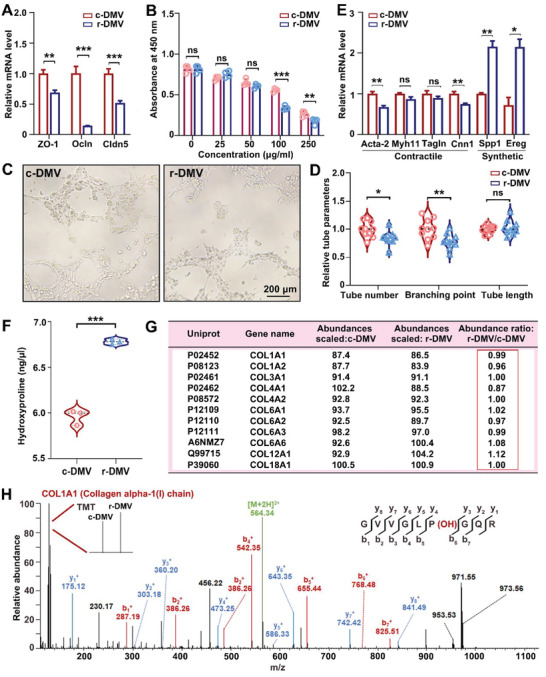
The r‐DMV‐derived EVs promote endothelial cell and smooth muscle cells dysfunction. A) Quantitative PCR analysis of tight junction proteins showing lower transcript levels in the r‐DMV group than in the c‐DMV group. *n* = 3 per group. B) EVs (100 µg mL^−1^ and 250 µg mL^−1^) from the r‐DMV group inhibited endothelial cell proliferation compared with the c‐DMV group. *n* = 4 per group. C) EVs from the r‐DMV group suppressed tube formation by bEnd.3 compared with the c‐DMV group. D) Quantification of tube number, branching point, and tube length to assess the tube formation ability. *n* = 10 per group. E) Quantitative PCR analysis of contractile and synthetic phenotype markers of smooth muscle cells treated with 50 µg mL^−1^ EVs for 48 h. *n* = 3 per group. F) Proline hydroxylation assay of serum EVs derived from c‐DMV (pooled samples of 119 participants) and r‐DMV (pooled samples of 118 participants). *n* = 3 per group. G) The abundance of collagen expressed in c‐DMV and r‐DMV derived EVs. H) Representative MS/MS spectral data of collagen proline hydroxylation peptide fragments. c‐DMV, control DMV; r‐DMV, reduced DMV. *P < 0.05, **P < 0.01, ***P < 0.001; ns, not significant. Student's *t*‐test was used as appropriate. Data represent the mean ± SEM.

Studies have shown that the blood‐brain barrier (BBB) leakage increases brain atrophy.^[^ ^26^ ^]^ The central elements of the BBB structure are tight junctions of endothelial cells. Therefore, we speculated that r‐DMV EVs may be involved in brain atrophy by affecting endothelial cell function. With tight junctions (TJ) playing a central role in BBB integrity, we first compared the levels of mRNA encoding BBB‐specific TJ proteins in bEnd.3 cells were treated with 100 µg serum‐derived EVs from the r‐DMV or c‐DMV groups. Compared with cells treated with c‐DMV EVs group, ZO‐1, Ocln, and Cldn5 were downregulated in the cells treated with r‐DMV EVs (**Figure** **5**A). Furthermore, CCK‐8 assays revealed prominent suppression of bEnd.3 cell growth following treatment with r‐DMV EVs at 100 and 250 µg mL^−1^, whereas there were no significant differences in the growth patterns of cells treated with r‐DMV EVs at 25 and 50 µg mL^−1^ (Figure 5B). The in vitro tube formation assay showed the ability of endothelial cells to form tube‐like structures reflecting the potential for cell migration and the establishment of cell‐cell and cell‐matrix contractions. To assess the effect of r‐DMV EVs on endothelial tube formation, bEnd.3 cells were treated with 100 µg mL^−1^ serum‐derived EVs from the r‐DMV or c‐DMV groups. bEnd.3 cells cultured for 6 h on gelled Matrigel containing s‐DMV EVs formed more tubule‐like networks than cells cultured in the presence of r‐DMV EVs (Figure 5C). These data showed that the tube number and branching of endothelial cells were significantly lower following treatment with r‐DMV EVs than after treatment with c‐DMV EVs, although there was no significant difference in tube length between the two groups (Figure 5D). Briefly, our data show that the r‐DMV EVs inhibited endothelial cell migrating capabilities.

VC is a pathological phenomenon caused by abnormal deposition of collagen in the vessel wall. Since collagen is known to increase in synthetic smooth muscle cells,^[^ ^27^ ^]^ we investigated whether the reduction in DMVs was due to increased collagen secretion by smooth muscle cells, resulting in abnormal deposition on the vessel wall. After treating rat thoracic aortic smooth muscle cells (A‐10) with different concentrations of EVs (50 µg mL^−1^, 100 µg mL^−1^, and 200 µg mL^−1^) from the c‐DMV and r‐DMV groups for 48 h, we observed lower levels of contractile markers (Acta‐2, Myh11, Tag1n, and Cnn1) and higher levels of synthetic markers (Spp1 and Ereg) in the r‐DMV group than those in the c‐DMV group (Figure 5E and S2, Supporting Information).

Hydroxyproline, which is required for proline hydroxylation as a post‐translational modification,^[^ ^28^ ^]^ is found in few proteins other than collagen and provides stability to the triple‐helical structure by forming hydrogen bonds. Analysis of the levels of hydroxyproline in the pooled serum‐derived EVs from the c‐DMV (*n* = 119) and r‐DMV (*n* = 118) groups revealed that the levels of hydroxyproline in the r‐DMV EVs were higher than those in the c‐DMV EVs (Figure 5F). According to our proteomic profiles, there was no difference in the collagen protein abundance between the c‐DMV EVs and r‐DMV EVs (Figure 5G) whereas we detected more collagen hydroxyproline in r‐DMV EVs than in c‐DMV EVs (Figure 5H). Thus, our results indicate that r‐DMV EVs switch smooth muscle cells to synthetic phenotypes and yield more collagen than c‐DMVs. Moreover, serum EVs are derived from r‐DMVs, the collagen of which may be more stable.

### 2.6 Quantitative Characteristics of Protein Profiles

**Figure 6:**
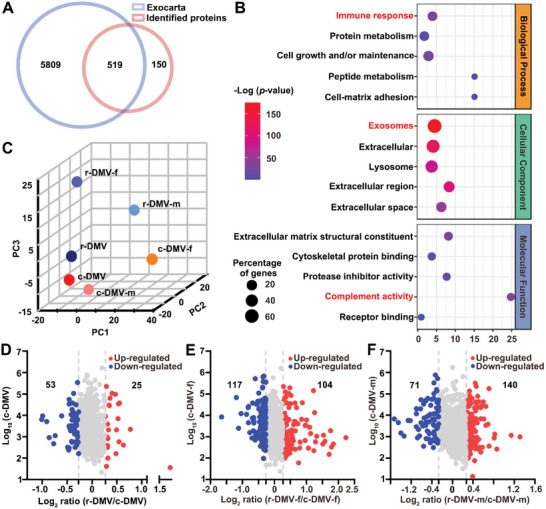
Quantitative characteristics of protein profiles. A) Venn diagram showing the overlap of identified proteins with ExoCarta proteins (519; Released date: 29 July 2015). B) The five most enriched categories and the enriched significance (‐log (*P*‐value), *P* < 0.05) of identified proteins in the cellular components, biological process, and molecular functions categories. C) Principal component analysis (PCA) showing differences in the proteome profiles between c‐DMV, r‐DMV, c‐DMV‐f, r‐DMV‐f, c‐DMV‐m, and r‐DMV‐m. D‐F) Scatter plot showing the distribution of upregulated (red dots) and downregulated (blue dots) differentially expressed proteins (DEPs). DEPs in r‐DMV and c‐DMV (D); DEPs in r‐DMV‐f and c‐DMV‐f (E); DEPs in r‐DMV‐m and c‐DMV‐m (F). c‐DMV, control DMV; r‐DMV, reduced DMV; c‐DMV‐f, females from control DMV group; r‐DMV‐f, females from reduced DMV group; c‐DMV‐m, males from control DMV group; and r‐DMV‐m, males from reduced DMV group.

To understand the functional role of EVs in cerebral venous disease‐associated brain atrophy, we investigated differences in the composition of serum‐derived EV proteins between the r‐DMV and the c‐DMV groups using TMT‐based quantitative MS. A schematic diagram of the experimental workflow is shown in Figure S3, Supporting Information. A total of 675 proteins (unique peptide ≥2 and false discovery rate <0.01) were identified (Table S2, Supporting Information). Among the identified EV proteins, 519 proteins (77.6%) overlapped with ExoCarta protein list (http://www.exocarta.org, release date: July 29, 2015) (**Figure** **6**A). Gene Ontology (GO) classification indicated that most of the identified EV proteins were derived from exosomes, with molecular functions in complement activity as well as the biological processes of immune response, protein metabolism, and cell growth and/or maintenance (Figure 6B). These categories were consistent with the reported functions of EVs (a complete list of all enriched GO terms is shown in Table S3, Supporting Information). Principal component analysis (PCA) of the proteomic profiles showed a clear separation between the r‐DMV and c‐DMV groups, confirming the distinct differences between the two types of serum‐derived EVs (Figure 6C). With a 1.2‐fold change cut‐off, 78 (25 upregulated and 53 downregulated) differentially expressed proteins (DEPs) were identified in the r‐DMV group compared with the c‐DMV group (Figure 6D), 221 DEPs (104 upregulated and 117 downregulated) were identified in the r‐DMV‐f group compared with the c‐DMV‐f group (Figure 6E), and 211 DEPs (71 upregulated and 140 downregulated) were identified in the r‐DMV‐m group compared with the c‐DMV‐m group (Figure 6F). The details of the DEPs are listed in Table S4, Supporting Information.

### 2.7 C3 is Related to the Reduction of DMV, Especially in Females

**Figure 7:**
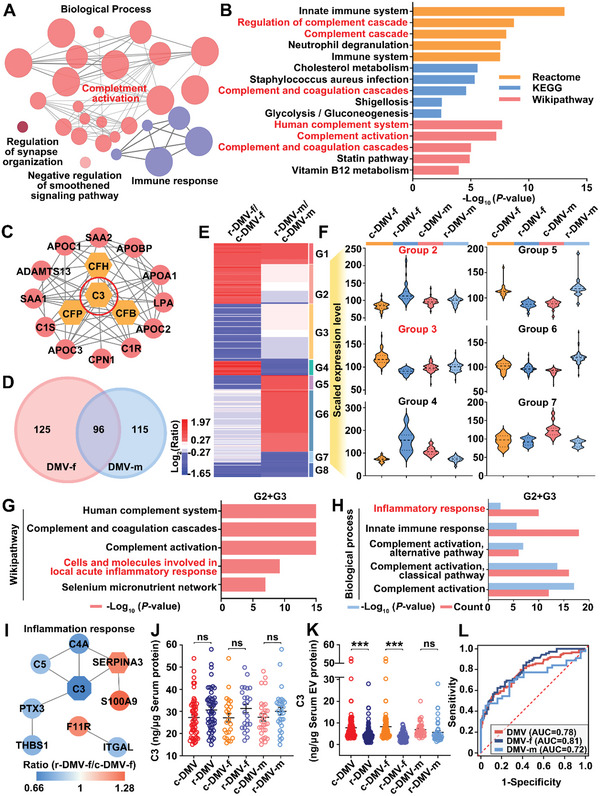
Protein profiles demonstrate that EV complement C3 is related to the reduced numbers of DMV, especially in females. A) GO analysis of the 78 differentially expressed proteins (DEPs) between the r‐DMV and c‐DMV groups. B) Reactome, KEGG, and WikiPathway analysis of the 78 DEPs. C) STRING analysis showing protein‐protein interactions of 78 DEPs between r‐DMV and c‐DMV groups with C3 as a hub protein. D) Venn diagram showing a total of 328 DEPs between DMV‐f (226 DEPs) and DMV‐m (195 DEPs). E) Heatmap of the 328 DEPs. G1–G8: Group1–Group8. F) The scaled expression level of c‐DMV‐f, r‐DMV‐f, c‐DMV‐m, and r‐DMV‐m. G) WikiPathway analysis of G2 and G3 DEPs. H) GO analysis of G2 and G3 DEPs. I) STRING analysis showing protein‐protein interactions of inflammation response‐associated proteins among 328 DEPs with C3 as a hub protein. J) Serum of participants with c‐DMV (*n* = 53), r‐DMV (*n* = 53), c‐DMV‐f (*n* = 24), r‐DMV‐f (*n* = 24), c‐DMV‐m (*n* = 29), and r‐DMV‐m (*n* = 29). K) Serum EVs of participants with c‐DMV (*n* = 119), r‐DMV (*n* = 118), c‐DMV‐f (*n* = 75), r‐DMV‐f (*n* = 75), c‐DMV‐m (*n* = 44), and r‐DMV‐m (*n* = 43). L) The diagnostic performances of EV C3, measured by AUC, were 0.78 (DMV), 0.81 (DMV‐f), and 0.72 (DMV‐m). c‐DMV, control DMV; r‐DMV, reduced DMV; c‐DMV‐f, females from control DMV group; r‐DMV‐f, females from reduced DMV group; c‐DMV‐m, males from control DMV group; and r‐DMV‐m, males from reduced DMV group. *P < 0.05, **`P < 0.01, ***P < 0.001; ns, not significant. Student's *t*‐test was used as appropriate. Data represent the mean ± SEM.

To clarify the mechanism by which r‐DMV EVs induce A1 reactive astrocytes and disrupted neuronal and endothelial cell function, we performed an in‐depth bioinformatics analysis of the proteomic profiles of serum‐derived EVs from the r‐DMV and c‐DMV groups, as well as their sex‐grouped EVs. When r‐DMV EVs were compared with c‐DMV EVs, the 78 DEPs between the r‐DMV EVs and c‐DMV EVs were significantly enriched in synapse organization, complement, and immune response (**Figure** **7**A). To gain insights into the potential functions of these DEPs, we employed the WEB‐based GEneSeTAnaLysis Toolkit (http://www.webgestalt.org/) to map the gene symbols of the DEPs to the Reactome, KEGG, and Wiki pathway databases. The 78 DEPs were predominantly enriched in the regulation of the complement cascade, complement and coagulation cascades, and human complement activation pathways (Figure 7B). To further place these DEPs in the context of known protein‐protein interactions and gain insights into the coordinated roles of these proteins, we investigated their potential interactions using the online resource STRING (https://string‐db.org/). Protein‐protein interaction networks were visualized using Cytoscape according to their STRING scores. Among these, complement C3 (C3) was a hub protein, and complement factor H (CFH), properdin (CFP), and complement factor B (CFB) were complement‐associated proteins (Figure 7C). In our previous analysis, we found that DMV‐reduction in females was more strongly associated with brain atrophy than in males (Figure 1C). We identified a total of 328 DEPs between DMV‐f (226 DEPs) and DMV‐m (195 DEPs), of which 93 were shared (Figure 7D), suggesting the existence of sex‐related differences between the serum‐derived EVs in males and females with DMV‐reduction. To further investigate the sex‐related differences among the EV proteins, we performed a cluster analysis (Figure 7E). Eight distinct protein clusters were identified based on fold changes in protein expression. In groups 1 and 8 (G1 and G8), these proteins showed similar expression trends in both female and male EVs groups. Groups 2 and 3 (G2 and G3) contained proteins that were DEPs in r‐DMV‐f compared with c‐DMV‐f, but not in r‐DMV‐m compared with c‐DMV‐m. In group 4 (G4), the proteins were upregulated in r‐DMV‐f and downregulated in r‐DMV‐m, whereas, in group 5 (G5), proteins were downregulated in r‐DMV‐f and upregulated in r‐DMV‐m. The proteins in groups 6 and 7 (G6 and G7) only showed changes in expression in the DMV‐f group, but not in the DMV‐m group. The DEPs in G1 and G8 were considered sex‐consistent, whereas those in G2–G7 were considered sex‐specific. Expression levels of sex‐specific DEPs in the female and male groups of r‐DMV and c‐DMV EVs are shown in Figure 7F. Among these, G2 and G3 were female‐specific DEPs. We further explored the potential function of female‐specific DEPs in DMV‐reduction. WikiPathway enrichment analysis indicated that in addition to the complement‐associated pathway, cells, and molecules involved in local acute inflammatory response also played a pivotal role (Figure 7G). Next, we clustered all the G2 and G3 proteins into GO categories using bioinformatics tools. In terms of biological process, the female‐specific DEPs were also enriched in inflammatory response (Figure 7H). Among the affected proteins involved in the inflammatory response term, C3 was downregulated and SERPINA3 was upregulated as hub proteins in the serum‐derived EVs of r‐DMV‐f compared with c‐DMV‐f (Figure 7I). We selected C3 and SERPINA3 for representative MS/MS identification, which yielded results that were consistent with the proteomic data (Figure S6, Supporting Information). C3 protein is the central component of the complement system, making it one of the most abundant proteins in circulation.^[^ ^29^ ^]^ To investigate the contribution of C3 to the clinical characteristics or progression of DMV‐reduction, we determined its levels in individual serum and EVs samples by ELISA. No significant differences were observed in serum C3 levels between the r‐DMV and c‐DMV groups. Additionally, there were no significant differences in the serum C3 levels of females and males of both the r‐DMV and c‐DMV groups (Figure 7J). We further detected the expression level of C3 in pooled serum EV samples from 119 c‐DMV (75 females and 44 males) and 118 r‐DMV (75 females and 43 males) participants. C3 levels were decreased in r‐DMV EVs and r‐DMV‐f EVs, whereas there was no significant difference between c‐DMV EVs and r‐DMV‐m EVs (Figure 7K). Further, ROC curve analysis of C3 expression levels in EVs from 119 c‐DMV samples and 118 r‐DMV samples showed that the performance of EV C3 expression levels for the diagnosis of DMV reduction was good, with AUC values of 0.78 (total), 0.81 (female), and 0.72 (male) (Figure 7L). These results suggested that C3 in EVs may serve as a biomarker for diagnosing DMV‐reduction and assessing brain atrophy.

## 3 Discussion

Although an association between DMV reduction and brain atrophy was described in our previous cohort study,^[^ ^5^ ^]^ the underlying mechanism is unclear. EVs cargo proteins have been identified as risk markers for the progression of vascular diseases and brain atrophy.^[^ ^9^ ^]^ In this study, we discovered that serum‐derived EVs from participants with DMV reduction promoted the loss of many normal neuronal functions, induced A1 reactive astrocytes, and caused endothelial and smooth muscle cell dysfunction more significantly than those derived from normal control participants. Proteomic profiles of these EVs were obtained by TMT‐based MS to clarify the mechanism of these cellular phenotypic changes and to further elucidate the role of EVs cargo proteins in brain atrophy. The DEPs identified in serum EV from r‐DMV participants were predicted to be involved in multiple processes and functions related to the complement system and inflammation, particularly in females. We hypothesized that serum‐derived EVs from r‐DMV participants promote brain atrophy by regulating inflammation‐related proteins, particularly by downregulating C3 and upregulating SERPINA3.

Our results demonstrated that serum EVs from participants with a reduced number of DMVs significantly impaired dendrite morphology and reduced the synaptic number and dendritic spine formation. Neuronal death and axonal destruction can result in brain atrophy,^[^ ^30^ ^]^ and we speculated that serum EVs may be involved in brain atrophy by affecting neuronal function. Thus, we used primary rat cortical neurons for in vitro functional studies and intravenous injection of EVs to mice for in vivo functional studies. DMV abnormalities have been reported in patients with mild cognitive impairment and early Alzheimer's disease.^[^ ^31^ ^]^ In Alzheimer's disease, the accumulation of Aβ peptides gradually leads to the hyperphosphorylation of tau proteins, which then form neurofibrillary tangles, resulting in brain atrophy. Our data showed that serum EVs significantly promoted the production of Aβ42 in the SH‐SY5Y cell line (Figure 3G) but had no obvious effect on the phosphorylation of Tau‐S396 (Figure 3H). Further studies of the functional effects of serum EVs on human‐derived neurons are required to validate our findings.

Astrocytes, which are abundant in the CNS,^[^ ^32^ ^]^ shape the microarchitecture of the brain, form neuronal‐glial‐vascular units, and regulate the BBB.^[^ ^33^ ^]^ Given the vital roles of astrocytes, it is not surprising that impairment of these functions has been implicated in the pathogenesis of many diseases. Particularly, A1 astrocytes, which are highly neurotoxic, have been linked to neurodegenerative diseases, including Alzheimer's disease.^[^ ^34^ ^]^ Therefore, we investigated the ability of serum EVs from participants with reduced DMV levels to change the reactive state of astrocytes. Our data revealed that the serum EVs from DMV‐reduction participants induced the upregulation of A1 reactive astrocytes. Based on these findings, we speculate that serum EVs from participants with DMV‐reduction participate in brain atrophy by inducing A1 reactive astrocyte‐mediated neurotoxicity.

BBB permeability, which is associated with brain atrophy, is regulated by tight junction proteins in brain endothelial cells.^[^ ^26^, ^35^ ^]^ Therefore, the expression and function of brain endothelial tight junction proteins, including occludin, claudins, and members of the membrane‐associated guanylate kinase protein family such as ZO‐1, are often used as metrics to assess BBB integrity. Claudin‐5 and occludin are transmembrane proteins, while ZO‐1 is a peripheral membrane protein. The loss of claudins and ZO‐1 is associated with BBB breakdown in human neurodegenerative disorders.^[^ ^36^, ^37^ ^]^ We speculated that EVs may be involved in the brain atrophy of participants with reduced DMV by affecting the function of endothelial cells. In accordance with this hypothesis, we found that the mRNA expression levels of claudin‐5, occludin, and ZO‐1 were decreased in the r‐DMV EV‐treated endothelial cells, suggesting that r‐DMV derived EVs perturb the tight junctions of endothelial cells. Vascular defects are associated with decreased endothelial cell proliferation and tube formation ability.^[^ ^38^ ^]^ Based on our findings, it is conceivable that r‐DMV serum‐derived EVs participate in the process of brain atrophy by inhibiting the proliferation, tube formation, and tight junction function of endothelial cells.

Under physiological conditions, vascular smooth muscle cells are contractile and maintain the elasticity of blood vessel walls and regular blood flow. However, under pathological conditions, these cells can dedifferentiate into a synthetic phenotype associated with the secretion of EV, proliferation, and migration to repair injury.^[^ ^39^ ^]^ This process, known as phenotypic switching, is often the first step in vascular pathology.^[^ ^40^ ^]^ In this study, we demonstrated that the mRNA levels of contractile markers were downregulated in smooth muscle cells after treatment with r‐DMV EVs, while the expression of synthetic markers was upregulated, indicating a switch to the synthetic phenotype. Therefore, we speculate the decreased number of DMVs in r‐DMV participants might be caused by increased synthesis of collagen, causing venous stenosis and obstruction. Regulation of HYP sites within the collagen structure alters fiber conformation and integrity and protects the collagen structure from degeneration.^[^ ^28^ ^]^ According to our proteomics profiles, proline hydroxylation of collagen was increased in r‐DMV EVs, indicating that the collagen in the r‐DMV group was more likely to be deposited in the venous walls and appeared as a reduction of DMVs in SWI.

To explore the mechanism underlying the function of r‐DMV EVs, we compared the proteomic profiles of serum‐derived EVs from participants in the r‐DMV and c‐DMV groups, as well as the corresponding groups divided according to sex. We found that multiple complement‐associated proteins were significantly enriched in the EVs of the r‐DMV group. Activation of the complement pathway leads to neuronal damage in various neurological diseases^[^ ^41^ ^]^ and can contribute to neurodegenerative phenotypes and brain atrophy.^[^ ^42^ ^]^ Among these complement‐associated proteins, we focused on C3 and SERPINA3 protein as the two proteins that were identified as female‐specific DEPs. As a central component of the complement system, C3 protein has been identified as a plasma biomarker of brain atrophy in Alzheimer's disease.^[^ ^43^ ^]^ In this study, we found that C3 protein was downregulated in EVs from r‐DMV participants compared with the c‐DMV group. It must be noted that very few studies have directly addressed the associations between serum‐derived EVs of complement‐associated proteins and their levels in the CNS. Interestingly, EVs from the r‐DMV group promoted C3 mRNA expression in astrocytes to a greater extent than EVs from the c‐DMV group. We speculate that there may be proteins other than C3 in the EVs from r‐DMV group that induce A1 reactive astrocytes. SERPINA3 is a glycoprotein belonging to the serine protease inhibitor family of acute phase proteins^[^ ^44^ ^]^ and was found to be markedly upregulated in the serum‐derived EVs from r‐DMV participants. SERPINA3 has been reported to be involved in the complement cascade and inflammation.^[^ ^44^ ^]^ Although the basal level of SERPINA3 expression in the brain is low, it is upregulated in activated astrocytes. Several studies have shown a correlation between increased level of circulating SERPINA3 and brain microbleeds in elderly patients.^[^ ^45^ ^]^ These data indicate that peripherally derived SERPINA3 directly influences the brain. Notably, the EV C3 levels were lower in the r‐DMV group than in the c‐DMV group, particularly in females. AUC values of 0.78 in r‐DMV and 0.81 in r‐DMV‐f, indicate that serum EV C3 expression levels can be used to discriminate individuals with DMV‐reduction from those with normal numbers of DMVs without the need for imaging to reduce the cost and not limited by instruments.

Changes in EVs may be a common upstream mechanism leading to both reduced deep medullary veins and brain atrophy. The findings of this study revealed that EVs from individuals with r‐DMVs play a role in the impairment of brain vascular cells and neurons. Additionally, in vivo experiments have demonstrated that r‐DMV EV induces abnormal neuronal activity and apoptosis and activation of astrocytes in mice. This phenomenon can be attributed to the cargo carried by EVs, which can transport diverse bioactive molecules, thereby influencing the functionality of the target cells.^[^ ^7^ ^]^ Additionally, we observed significant differences in the protein composition and EV content between individuals with r‐DMVs and those with c‐DMVs. These findings suggest that r‐DMV EVs may provoke pathological alterations by inflicting damage on brain vascular cells, releasing EVs that can enter the local brain tissue or bloodstream, and perpetuating a detrimental cycle that exacerbates brain injury and contributes to the onset and progression of brain atrophy. Thus, the relationship between r‐DMV and atrophy is complex and the EV may play a role in this network and whether it could be developed as a therapeutical target or bio‐marker awaits further investigation.

The limitations of our study should also be considered. This study was based on an ongoing prospective community‐based cohort; therefore, our findings must be reproduced using other samples. Further, the absence of a suitable model for brain atrophy hinders further in‐depth research into this condition. While animal models can provide some understanding regarding brain atrophy to some extent, they are unable to fully replicate its complexity. Nevertheless, we elucidated the proteomic characteristics of serum‐derived EVs in c‐DMV and r‐DMV participants, and our findings expand the current knowledge of the roles of EVs in the cerebral DMV reduction‐associated brain atrophy.

## 4 Conclusion

**Figure 8:**
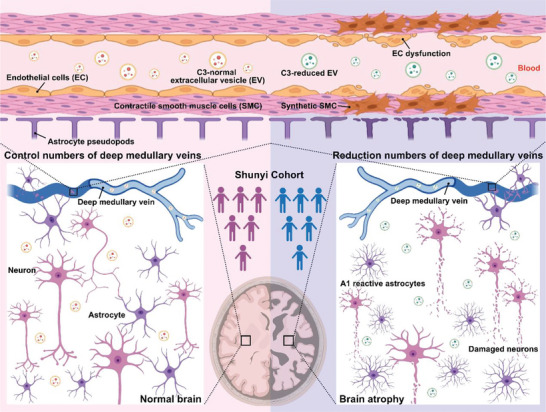
Graphical abstract summarizing the results.

In summary (**Figure** **8**), serum‐derived r‐DMV EVs promote the dysfunction of neurons, endothelial cells, and smooth muscle cells and induce A1 reactive astrocytes. These phenotypes are associated with brain atrophy. The expression profile of the serum EV proteome in r‐DMVs differed from that of c‐DMVs. This difference may be attributed to changes in complement‐related proteins, particularly C3. Furthermore, we identified C3 as a potential biomarker of DMV reduction.

## 5 Experimental Section

### Reagents

All antibodies, commercial assays, and media in this study are listed in Table S5, Supporting Information.

### Study Population

The study population was selected from the Shunyi Study, a prospective population‐based cohort study on age‐related cardiovascular and neurological diseases.^[^ ^46^ ^]^ Between June 2013 and April 2016, 1586 inhabitants in Shunyi, Beijing aged ≥35 years were recruited, of which 1257 underwent magnetic resonance imaging (MRI) examination. After excluding individuals with SWI artifacts and severe defects that hindered DMV measurement (large periventricular infarcts or hemosiderin deposition, or enlarged lateral ventricles) (*n* = 167), those with a history of stroke (*n* = 34), and those with poor image quality (*n* = 77), a total of 979 participants were included for DMV measurement.^[^ ^5^ ^]^ After sorting participants according to DMV number, those in the first quartile (DMV number ≤ 17.5, *n* = 220) in the DMV‐reduction (r‐DMV) group and those in the last quartile (DMV number ≥ 20, *n* = 327) in the control (c‐DMV) group was included. To control for confounding factors, the age and sex of each participant in the two groups are matched, and included only participants aged between 57 and 79 years, leaving 124 participants in each group. Due to concerns regarding the acquisition and preservation of serum samples, it was decided to use serum preserved in 2015 and excluded participants without serum samples or with hemolytic serum. Finally, 118 and 119 participants were included in the r‐DMV and c‐DMV group, respectively (Figure S5, Supporting Information).

### DMV Measurement

DMV measurements were performed as a previously described method.^[^ ^6^ ^]^ Briefly, DMVs were counted in both hemispheres and the average number was recorded as the final DMV number (Figure S6, Supporting Information).

### Clinical Data

All participants underwent baseline clinical assessments and were invited to undergo annual follow‐ups. Clinical information including age, sex, body mass index (BMI), and history of hypertension, diabetes mellitus, and hyperlipidemia was collected at baseline using both questionnaires and physical examinations. Cognitive status was evaluated by MMSE and MOCA scores. MRI markers were evaluated using T1‐weighted, T2‐weighted, fluid‐attenuated inversion recovery (FLAIR), and SWI results were obtained with a 3‐T Skyra scanner (Siemens, Erlangen, Germany). Imaging markers of CSVD including lacunae, cerebral microbleeds (CMBs), and white matter hyperintensities (WMH) were defined according to the Standards for Reporting Vascular Changes on Neuroimaging.^[^ ^47^ ^]^ Silent brain infarcts (SBIs) were defined as hypointense lesions (3–20 mm diameter) in T1 MRI and hyperintense lesions in T2 and FLAIR MRI. BPF is considered an indicator of brain atrophy. The detailed calculation methods have been published elsewhere.^[^ ^5^ ^]^ Hemoglobin and hypersensitive C‐reactive protein (hsCRP) levels were measured in overnight fasting venous blood within 3 h of collection at the Department of Clinical Laboratory of Peking Union Medical College Hospital.

This study was approved by the Ethics Committee of Peking Union Medical College Hospital (reference number: B‐160) and conducted in accordance with the principles of the Declaration of Helsinki. All participants provided written informed consent.

### Isolation of Serum‐Derived Extracellular Vesicles (EVs)

Serum‐derived EVs were isolated by size exclusion chromatography (qEV original/35 nm, ICO‐35, IZON Science) according to the manufacturer's protocol. Equal amounts of individual serum samples in each group were pooled to isolate the EVs. For each group, 400 µL serum sample was then centrifuged at 1500×*g* for 10 min to remove any cells and large particles. Subsequently, the supernatant was centrifuged at 10000×*g* for 10 min and loaded onto qEV columns, and EVs were collected as fractions, which were then concentrated using an Amicon Ultra‐4 30 K centrifugal filter unit (UFC8030, Merck). The EV suspension was lysed with RIPA lysis buffer or 8 M urea and the protein concentration was determined using a bicinchoninic acid (BCA) protein assay kit (23 225, Thermo Fisher Scientific) according to the manufacturer's instructions. The EVs were characterized by electron microscopy, nanoparticle tracking analysis, and western blot analysis of the EV markers LAMP2, ALIX, and CD63, with ALB as a negative marker.

### Coomassie Blue Staining and Western Blot Analysis

For the EV proteomics, equal amounts of proteins (30 µg) from EV lysates were separated using 12% sodium‐dodecyl sulfate‐polyacrylamide gel electrophoresis (SDS‐PAGE) for Coomassie blue staining.

For the cell assays, SH‐SY5Y cell pellets or EVs were collected and lysed in RIPA supplemented with Complete Protease Inhibitor Cocktail (11 697 498 001, Roche). Total protein concentrations of samples were determined using bicinchoninic acid (BCA) protein assay kits (23 225, Thermo Fisher Scientific). Equal amounts of total protein (30 µg) were separated using 10% SDS‐PAGE and transferred onto polyvinylidene fluoride (PVDF) membranes. After being blocked with 5% nonfat dry milk in Tris Buffered Saline plus 0.5% Tween (TBST), the membranes were incubated with the following primary antibodies overnight at 4 ^ο^C:anti‐LAMP2 (1:1000, 27823‐1‐AP, Proteintech), anti‐ALIX (1:1000, ab186429, Abcam), anti‐CD63 (1:1000, sc‐15363, Santa Cruz), anti‐ALB (1:1000, ab10241, Abcam), anti‐CD144 (1:1000, 2500, Cell Signaling Technology), anti‐PDGFRβ (1:5000, ab32570, Abcam), anti‐Myh11 (1:1000, ab133567, Abcam), anti‐phosphoTau (ser396) (1:1000, 44–752G, Thermo Fisher Scientific), anti‐Tau (1:1000, ab32057, Abcam), and anti‐ACTB (1:10000, GTX124213, GenTex) as a loading control. After incubation with the horseradish peroxidase (HRP)‐conjugated secondary antibodies (1:10000) for 1 h at room temperature, protein bands were detected using Immobilon Western Chemiluminescent HRP Substrate (WBKLS0500, Millipore) and visualized using ChemiDoc XRS+ with Image Lab software (BioRad).

### EVs Flow Cytometry

Based on particle concentration measurements obtained by NTA, the volume of the sample stained represented ≈1 × 10^10^ particles in a final volume of 500 µL with 0.15 M filtered PBS. Prior to data collection, all samples were diluted to a final concentration of 1 × 10^8^ particles mL^−1^ in 0.15 M filtered PBS.

EVs in stained serum samples were quantified by flow cytometry using a CytoFLEX (Beckman Coulter) configured to Violet (405 nm) side scatter (SSC) as the trigger parameter, with the threshold set as 2000. The V‐SSC detector gain was set at 400 and FITC fluorescence was measured using a 525/40 nm bandpass filter with a detector gain set at 164; settings were chosen based on general guidelines for threshold and gain setting and on balancing between minimizing noise and using 0.15 M filtered PBS as a background control. FITC was chosen as the fluorophore for all markers based on its size, brightness, and ability to separate cleanly from background. Samples were recorded for 1000000 particles at the slow flow rate (10 µL min^−1^). FITC positively was determined by setting gates based on the unstained serum sample, and the stained samples stained with anti‐CD144 (1:100, 2500, Cell Signaling Technology), anti‐PDGFRβ (1:20, ab32570, Abcam), anti‐Myh11 (1:20, ab133567, Abcam) for 1 h at room temperature. Then, incubated with fluorescent secondary antibodies goat anti‐rabbit IgG Alexa Fluor 488 (1:400, A11034, ThermoFisher) for 30 min at room temperature. The EV gate was set independently for each serum sample and remained constant for the analysis of all cell markers.

### Primary Culture of Neurons and Astrocytes

Sprague–Dawley rats were obtained from Beijing HFK Bio‐Technology Corporation. All animal experiments were performed in accordance with the protocol approved by the Ethics Committee of the Institute of Basic Medical Sciences, China. Tissue was dispersed by 0.25% trypsin and 5 mg mL^−1^ DNase I digestion for 20 min at 37 °C. After digestion, the tissues were filtered with the 70 µm nylon mesh cell strainer. Neurons (3 × 10^5^cells per 24‐wellplate) were grown on poly‐D‐lysine‐coated glass coverslips in Neurobasal Medium Plus supplemented with B27, glutamine, and penicillin‐streptomycin; half of the medium was replaced every 2–3 days and cells were used on the day in vitro day (DIV) 5–7. Cortical astrocytes were prepared from postnatal day 1 rats. Briefly, the rat cortices were enzymatically digested (0.25% trypsin) at 37 °C for 15 min. The supernatant containing dissociated cells was passed through a 70 µm nylon mesh cell strainer into a 50 mL conical tube and centrifuged at 200 ×*g* for 5 min to pellet the cells. The supernatant was removed, and the cells were resuspended in Dulbecco's modified Eagle's medium (DMEM) containing 20% fetal bovine serum and 1% penicillin‐streptomycin and plated in 10 cm dishes.

### Cell Culture

The human neuroblastoma cell line (SH‐SY5Y) was cultured in RPMI 1640 medium (modified without Calcium Nitrate, with L‐glutamine, SH30809.01, HyClone) with inactivated 15% FBS. The mouse brain vascular endothelial cell line (bEnd.3) was cultured in RPMI1640 medium (containing 25 mmol L^−1^ HEPES and L‐glutamine, SH30255.01, HyClone) with 10% fetal bovine serum (10099‐141C, Gibco). Rat thoracic aortic smooth muscle cells (A‐10) were maintained DMEM (SH30243.01, Hyclone) with 10% FBS. Rat primary neurons were cultured in neurobasal medium (21 103 049, Thermo) with 2% B‐27 (12587‐010, Gibco), 0.5 mM L‐glutamine (SH15140‐122, Gibco), and 0.5% penicillin‐streptomycin (15140‐122, Gibco). Rat primary astrocytes were cultured in DMEM supplemented with 20% FBS and 1% penicillin‐streptomycin. All cells were cultured at 37 °C in a humidified atmosphere containing 5% CO_2_. At 80% confluence, the cells were trypsinized and suspended in a fresh medium.

### EV Staining with PKH26 Dye

Extracellular vesicles were derived from 1 mL serum per experimental group using size exclusion chromatography (qEV original/35 nm, ICO‐35, IZON Science) as previously described. The resulting vesicles were concentrated using an Amicon Ultra‐4 30K centrifugal filter unit (UFC8030, Merck). A total of 1 mL of concentrated EVs, containing ≈800 µg of protein (determined by BCA test), were subjected to staining with PKH26 using PKH26 Red Fluorescent Cell Linker Kits for General Cell Membrane Labelling (MINI26, Sigma‐Aldrich). The manufacturer's instructions were followed, whereby EVs were incubated with PKH26 at 37 °C for 10 min. The reaction was then terminated using 1% BSA. Subsequently, the labeled EVs were centrifuged at 200 000 g for 90 min at 4 °C to remove excess dye and resuspended in PBS buffer for in vivo imaging.

### Biodistribution of EVs in Mice

EVs labeled with PKH26 dye were injected intravenously into 4‐week‐old female C57/BL6 mice at a dose equivalent to 23 mg kg^−1^ body weight. After a period of 72 h postinjection, the mice were euthanized. The organs of interest, including the brain, heart, liver, lung, kidney, and spleen were fixed in 4% paraformaldehyde, followed by gradient dehydration in 20%−30% sucrose solution, embedding in O.C.T compound and stored at −80 °C for subsequent immunofluorescence staining.

### Immunofluorescence Staining

For in vitro assays, rat primary neurons and astrocytes were cultured on coverslip pre‐coated with poly‐lysine. For in vivo assays, the frozen organs were cut at a thickness of 12 µm sections. The cells on a coverslip and the tissue sections were fixed in 4% PFA for 30 min, and washed three times with PBS. After permeabilizing with 3% Triton X‐100 for 10 min, the cells were blocked with 5% BSA for 30 min and then incubated overnight at 4 °C with primary antibodies against MAP2 (1:200, ab254143, Abcam), synapsin I (1:200, ab254349, Abcam), PSD95 (1:200, 124 011, Synaptic Systems), NeuN (1:200, ab177487, Abcam), Cleaved Caspase 3 (1:200, 9661, Cell Signaling Technology) and Alexa Fluor 488‐Phalloidin (1:200, A12379, Thermo Fisher Scientific). After washing with PBS, fluorescent secondary antibodies goat anti‐mouse IgG Alexa Fluor 488 (1:200, A32723, Thermo Fisher), goat anti‐rabbit IgG Alexa Fluor 594 (1:200, A11037, Thermo Fisher Scientific), goat anti‐rabbit IgG Alexa Fluor 488 (1:200, A11034, Thermo Fisher Scientific), and goat anti‐mouse IgG Alexa Fluor 594 (1:200, A11032, Thermo Fisher Scientific) were added for 1 h at room temperature. Cells were stained with DAPI to visualize the nuclei. The total dendrite length was measured for primary dendrites and all dendritic branches. For neuronal morphology, the number of intersections was assessed by Sholl analysis.^[^ ^48^ ^]^ All measurements and 3D reconstruction of neurons were performed using the Imaris software.

### Quantitative Real‐Time PCR (RT‐qPCR)

Total RNA was extracted from cultured cells using TRIzol reagent (15 596 026, Thermo Fisher Scientific) according to the manufacturer's instructions. Extracted RNA (500 ng) was then used as a template for cDNA synthesis in 10 µL volume with the PrimeScript RT reagent kit (RR036A, TaKaRa). Quantitative real‐time PCR was performed using cDNA in 20 µL reaction with 10 µL TB Green Premix Ex Taq II (Tli RNase H Plus). Thermocycling conditions were 95 ^ο^C for 30 s, followed by 40 cycles at 95 ^ο^C for 5 s, 60 ^ο^C for 30 s, and 95 ^ο^C for 10 s. β‐actin and GAPDH served as the reference gene and relative gene expression was calculated with the 2^−ΔΔ^ *^CT^* method.^[^ ^49^ ^]^ The primer sequences are shown in Table S6, Supporting Information.

### Synaptosome Engulfment Assay

Synaptosomes were isolated from rat brain cortex using the Synaptic Extraction Reagent (Syn‐PER Synaptic Protein Extraction Reagent, 87 793, Thermo Fisher Scientific) according to the manufacturer's instructions and conjugated with pHrodo Red, succinimidyl ester (P36600, Thermo Fisher Scientific) at room temperature with gentle agitation. After incubation for 2 h, unbound pHrodo was removed by centrifugation. Astrocytes were then incubated with pHrodo‐conjugated synaptosomes in glial medium with c‐DMV or r‐DMV EVs for 48 h and imaged using a Leica time‐lapse microscope. For image analysis, we used a 20× objective lens to select 10 images per group from random areas of a 24‐well plate and plotted the total sum of the fluorescence intensity of the objects in the image. Data were normalized to the degree of engulfment per image.

### ELISA

Cell pellets and serum‐derived EVs were lysed in RIPA buffer and total proteins were extracted by sonication. C3 levels were detected using an ELISA kit (CSB‐E08665h, Cusabio). After treatment with EVs from r‐DMV and c‐DMV, the level of soluble Aβ1‐40 and Aβ1‐42 in SH‐SY5Y cell pellets were detected using Aβ1‐40 (CSB‐E08299 h, Cusabio) and Aβ1‐42 (CSB‐E10684 h, Cusabio) ELISA kits. All kits were used according to the manufacturer's instructions.

### Cell Proliferation Assay

bEnd.3 cells were seeded in 96‐well plates (1800 cells per well) and incubated in serum‐free medium for 2 h at 37 °C. EVs were then added to stimulate cells for 1 h before the medium was replaced with a medium containing 10% FBS. After incubation for 72 h, 10 µL of CCK8 (HY‐K0301, MCE) was added into each well and the cells were incubated for an additional 2 h before cell growth was determined by measuring the absorbance at 450 nm using Multiskan FC Microplate Photometer (Thermo Fisher Scientific).

### Tube Formation Assay

The formation of capillary‐like structures was determined using the basement membrane extract (Corning Matrigel Growth Factor Reduced Basement Membrane Matrix, CLS354230). After thawing at 4 °C, the Matrigel was immediately layered into 96‐well cell culture plates and incubated at 37 °C for 30 min to allow the basement membrane to gel. Subsequently, bEnd.3 (4.5 × 10^3^ cells) containing 100 µg mL^−1^ serum‐derived EVs from the c‐DMV or r‐DMV groups were seeded onto the Matrigel. After incubation at 37 °C for 6 h, images were captured using bright‐field microscopy. The tube number, branching point, and tube length were quantified using the Wimasis Analysis Platform (https://mywim.wimasis.com/).

### Hydroxyproline Assay

The hydroxyproline content of serum‐derived EVs was assessed using a hydroxyproline assay kit (MAK008, Sigma‐Aldrich) according to the manufacturer's protocol. Briefly, EVs isolated from 200 µL serum from the c‐DMV (*n* = 119) and r‐DMV (*n* = 118) groups were pooled and transferred to a pressure‐tight polypropylene vial containing 100 µL of concentrated hydrochloric acid and the PTFE‐lined cap was closed tightly. Aliquots (100 µL) of the solution were then transferred to a 96‐well plate and placed at 60 °C to dry samples. The amount of hydroxyproline in the samples was detected by a chromogenic reaction using chloramine‐T and 4‐dimethylaminobenzaldehyde as the substrates, and the absorbance was measured at 570 nm.

### Proteomic Analysis—Tandem Mass Tag (TMT) Labeling

For TMT labeling, the lysates of EVs from the six sample groups (c‐DMV, control DMV; r‐DMV, reduced DMV; c‐DMV‐f, females from control DMV group; r‐DMV‐f, females from reduced DMV group; c‐DMV‐m, males from control DMV group; and r‐DMV‐m, males from reduced DMV group) were diluted to 1 mg mL^−1^ with 8 M urea. The TMT kit (90 068, Thermo Fisher Scientific) was used for labeling according to the manufacturer's protocol. For each group, an aliquot of protein (100 µg) was reduced by incubation with 10 mmol L^−1^ dithiothreitol at 37 °C for 30 min, and then alkylated with 25 mmol L^−1^ iodoacetamide at room temperature for 30 min in the dark. Protein digestion was completed by incubation with Trypsin/Lys‐C (enzyme: protein mass ratio 1:25) for 14 h at 37 °C. After terminating the digestion by heating at 60 °C for 30 min, protein digests were desalted using reverse‐phase column cheomatography (Oasis HLB, WAT09425, Waters), dried with a SpeedVac vacuum concentrator, and finally dissolved in 200 mM triethylammonium bicarbonate buffer (pH 8.5) for labeling with 0.8 mg TMT reagents at room temperature. The groups were labelled as follows: c‐DMV, TMT‐126; r‐DMV, TMT‐127; c‐DMV‐f, TMT‐128; r‐DMV‐f, TMT‐129; c‐DMV‐m, TMT‐130; r‐DMV‐m, TMT‐131. After labeling, samples were mixed, desalted, dried as described previously, and finally dissolved in 0.1% formic acid for high‐performance liquid chromatography (HPLC) fractionation.

### Proteomic Analysis—High‐Performance Liquid Chromatography (HPLC) Fractionation

The TMT‐labelled protein digests (100 µL in 0.1% TFA) were fractionated by HPLC (UltiMate 3000 UHPLC, Thermo Scientific) equipped with an Xbridge BEH300 C18 column (4.6 mm × 250 mm, 5 µm) maintained at 40 °C with a flow rate of 1.0 mL min^−1^. Peptides were eluted using a gradient acetonitrile elution buffer consisting of H_2_O with ammonium hydroxide (mobile phase A, pH 10), and 98% acetonitrile with ammonium hydroxide (mobile phase B, pH 10). A total of 47 fractions were collected at 1.5 min intervals, dried in a vacuum concentrator, and combined into 12 samples according to the peptide abundance. The samples were dissolved in 20 µL of 0.1% TFA for subsequent liquid chromatography (LC)‐mass spectrometry with tandem mass spectrometry (LC‐MS/MS) analysis.

### Proteomic Analysis—LC‐MS/MS Analysis

The Ultiate U3000 system, directly connected to a Thermo Orbitrap Fusion Lumos mass spectrometer, was used for LC‐MS/MS analysis. The analytical column was a homemade fused silica capillary column (75 µm ID, 150 mm length; Upchurch, Oak Harbor, WA, USA) filled with C‐18 resin (300 Å, 2 µm; Varian, Lexington, MA, USA). The peptides were separated using a 135‐minute gradient elution with mobile phase A consisting of H_2_O with ammonium hydroxide (pH 10), and mobile phase B consisting of 98% acetonitrile with ammonium hydroxide (pH 10) at a flow rate of 0.3 µL min^−1^. Xcalibur 4.1 software was used to operate the Orbitrap Fusion mass spectrometer in the data‐dependent acquisition mode with a single full‐scan mass spectrum in the Orbitrap (350–1500 m/z, 120000 resolution) followed by 3‐s data‐dependent MS/MS scans in an ion routing multipole at 35% normalized higher‐energy collisional dissociation (HCD).

### Proteomic Analysis—Data Processing

MS/MS spectra were analyzed using Proteome Discoverer 2.4 software (Thermo Scientific). The spectra were searched against the UniProt/SwissProt human proteome database (released on August 11, 2022). The following parameters were set as the search criteria: full tryptic specificity with a maximum of two missed cleavages were allowed; precursor mass tolerance was set at 20 ppm; and fragment mass tolerance was set at 0.02 Da. Static modifications include carbamidomethylation on C and TMT 6‐plex on peptide N‐terminus. Dynamic modifications include TMT 6‐plex on K and oxidation on M. In the current study, identified proteins were defined as proteins with at least two unique peptides. The reported monoisotopic m/z was tuned according to the raw spectral data. The hydroxylated proline modification site localization probabilities were obtained by repeated searches of the MS/MS data using the UniProt/SwissProt human collagen database (released on August 23, 2022) and allowing for dynamic proline oxidation modification.

### Proteomic Analysis—Bioinformatics Analysis

For proteomic analysis of human serum‐derived EVs, the relative protein abundance was presented as the ratio of TMT‐127/126 (r‐DMV/c‐DMV group), TMT‐129/128 (r‐DMV‐f/c‐DMV‐f group), and TMT‐131/130 (r‐DMV‐m/c‐DMV‐m group). The differential expression threshold was defined as a 1.2‐fold change in expression. Details of the MS proteomic data were submitted to the ProteomeXchange Consortium with the identifier PXD 03 9873. GO functional enrichment analysis was conducted using the Database for Annotation, Visualization, and Integrated Discovery (DAVID) (https://david.ncifcrf.gov/) and FunRich software (3.1.3). GO analysis was performed using the Cytoscape plug‐in ClueGO (version 3.9.1). Pathway mapping was performed using the WEB‐based GEneSeT Toolkit (http://www.webgestalt.org/). Pathways with at least three target genes and a *P*‐value of <0.05 were considered significant. The STRING (Search Tool for the Retrieval of Interacting Genes/Proteins) database (https://string‐db.org/) was used to predict protein networks. Networks were downloaded and visualized using Cytoscape plug‐in MCODE (version 3.9.1).

### Statistical Analysis

For the proteome data, the proteins with missing values in all samples were removed. Stringent criteria were applied for high protein confidence (unique peptide ≥ 2) and contaminants from keratin proteins were eliminated. All the statistical analyses and data presentations were performed using R (version 4.1.0) and GraphPad Prism 8 software (GraphPad Software, Inc., La Jolla, CA, USA). All assays were repeated at least three times and the data were presented as mean ± standard error of mean (SEM). Unpaired two‐tailed Student's *t*‐test was used to compare the two experimental groups. *P*‐value < 0.05 was set as the threshold for statistical significance.

## Conflict of Interest

The authors declare no conflict of interest.

## Supporting information

Supporting Information

Supplemental Table 1

Supplemental Table 2

Supplemental Table 3

## Data Availability

The data that support the findings of this study are available in the supplementary material of this article.
