# Supplementary material for: Extracellular Vesicles Play a Central Role in Cerebral Venous Disease‐Associated Brain Atrophy
Source: Adv Sci (Weinh). 2023 Jul 12;10(27):2301574. doi: 10.1002/advs.202301574 (PMC10520650; doi:10.1002/advs.202301574)
Supplement: Supplementary file 1 — Supporting Information [file ADVS-10-2301574-s001.pdf]

ADVANCED SCIENCE
Open Access

Supporting Information

for *Adv. Sci.*, DOI 10.1002/advs.202301574

Extracellular Vesicles Play a Central Role in Cerebral Venous Disease-Associated Brain Atrophy

Jia-Yu Wang, Jing-Ying Li, Dan Luo, Mei-Ying Huang, Dong-Hui Ao, Xin-nan Liu, Xia Wang, Wei Ge and Yi-Cheng Zhu**

Supplementary Materials for

Extracellular vesicles play a central role in cerebral venous disease-associated brain atrophy

Jia-Yu Wang, Jing-Ying Li, Dan Luo, Mei-Ying Huang, Dong-Hui Ao, Xin-nan Liu, Xia Wang, Wei Ge*, Yi-Cheng Zhu*

*Email: gewei@ibms.cams.cn
zhuyc@pumch.cn

This file includes:

Figure S1. r-DMV EVs induce reactivity in A1-type astrocytes.
Figure S2. r-DMV EVs induce switching of smooth muscle cells from a contractile to a synthetic phenotype at the mRNA level.
Figure S3. Schematic workflow of the TMT-based quantitative proteomic analysis of extracellular vesicles (EVs) isolated from the pooled serum of volunteers with a reduction in the number of deep medullary veins (DMVs) and a control group.
Figure S4. Validation of the expression levels of DEPs by representative MS/MS.
Figure S5. Flowchart of information about the enrolled participants.
Figure S6. Images used for DMV assessment.

Table S1. Detailed information of 237 participants
Table S2. Identified proteins
Table S3. GO term of identified proteins
Table S4. Details of the DEPs
Table S5. Reagents and resources table
Table S6. Sequences of the primers

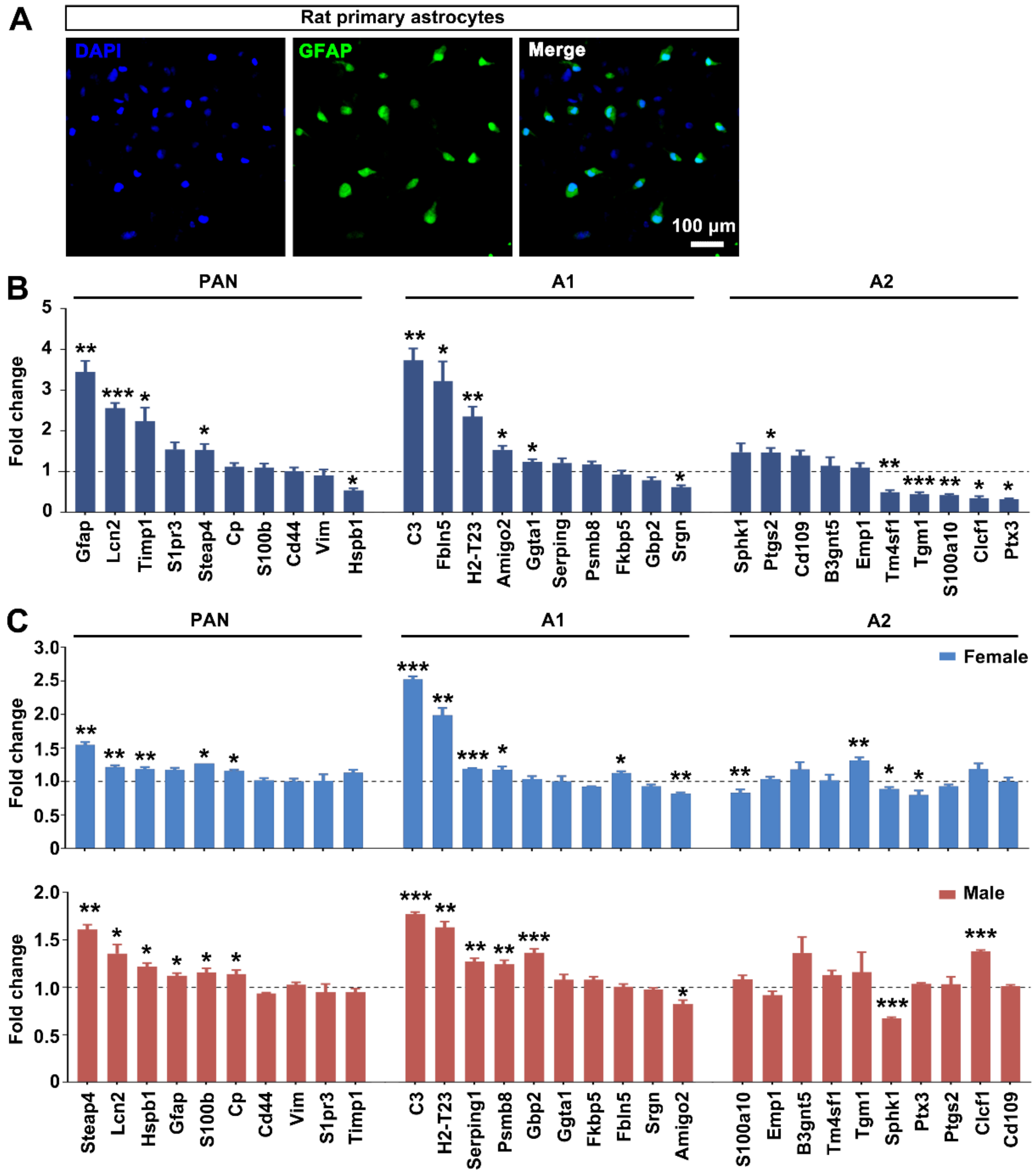

Figure S1. r-DMV EVs induce reactivity in A1-type astrocytes

(A) Representative confocal images of rat primary astrocytes immunostained with astrocyte marker GFAP (green) and DAPI (blue). (B) Quantitative PCR analysis of pan-reactive and A1- and A2- reactive transcripts in astrocytes treated with c-DMV and r-DMV EVs.; and (C) in astrocytes treated with c-DMV-f, r-DMV-f, c-DMV-m, and r-DMV-m EVs. **P* <0.05, ***P* <0.01, ****P* <0.001; ns, not significant. Student's *t*-test was used as appropriate. Data represent the mean ± SEM.

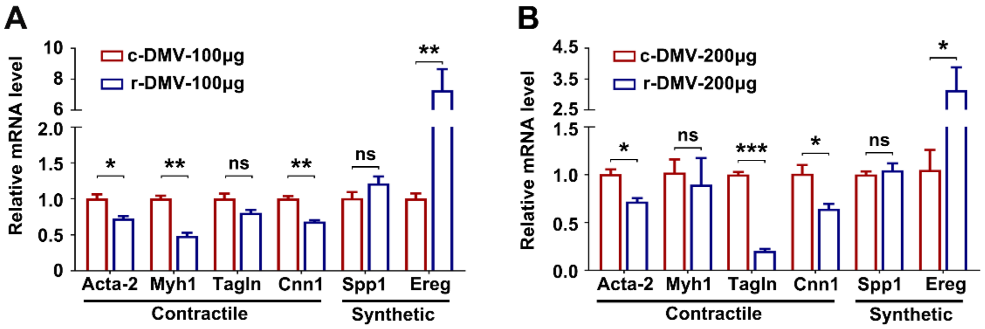

Figure S2. r-DMV EVs induce switching of smooth muscle cells from a contractile to a synthetic phenotype at the mRNA level

Smooth muscle cells (A-10) were treated with (A) 100 μg/mL and (B) 200 μg/mL EVs for 48 h.

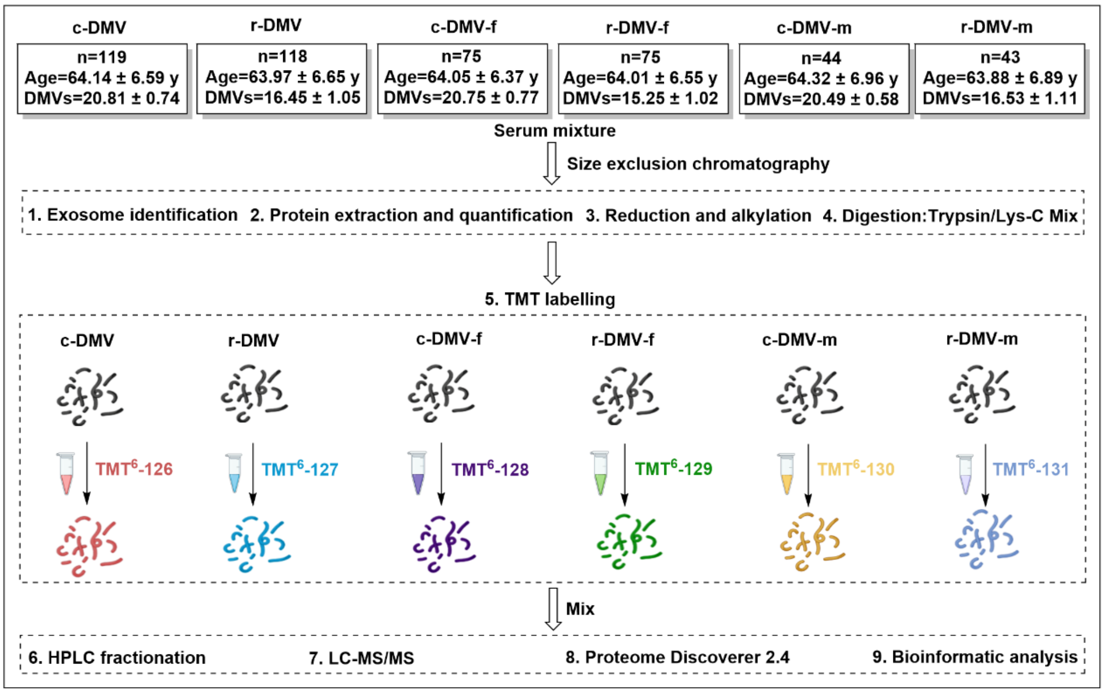

Figure S3. Schematic workflow of the TMT-based quantitative proteomic analysis of extracellular vesicles (EVs) isolated from the pooled serum of volunteers with a reduction in the number of deep medullary veins (DMVs) and a control group

c-DMV, control numbers of DMV; r-DMV, reduced numbers of DMV; c-DMV-f, females with control numbers of DMV; r-DMV-f, females with reduced numbers of DMV; c-DMV-m, males with control numbers of DMV; and r-DMV-m, males with reduced numbers of DMV. The original elements used in this diagram are from BioRender App (https://app.biorender.com/).

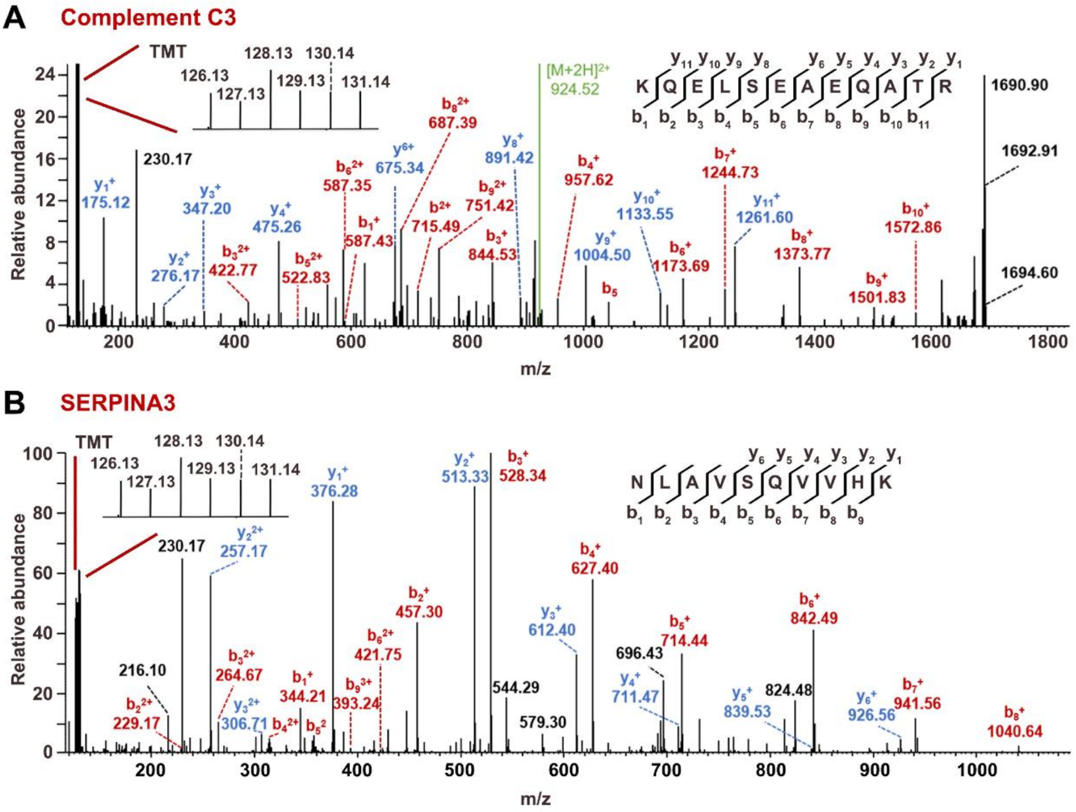

Figure S4. Validation of the expression levels of DEPs by representative MS/MS

Representative mass spectral data of C3 (A) and SERPINA3 (B).

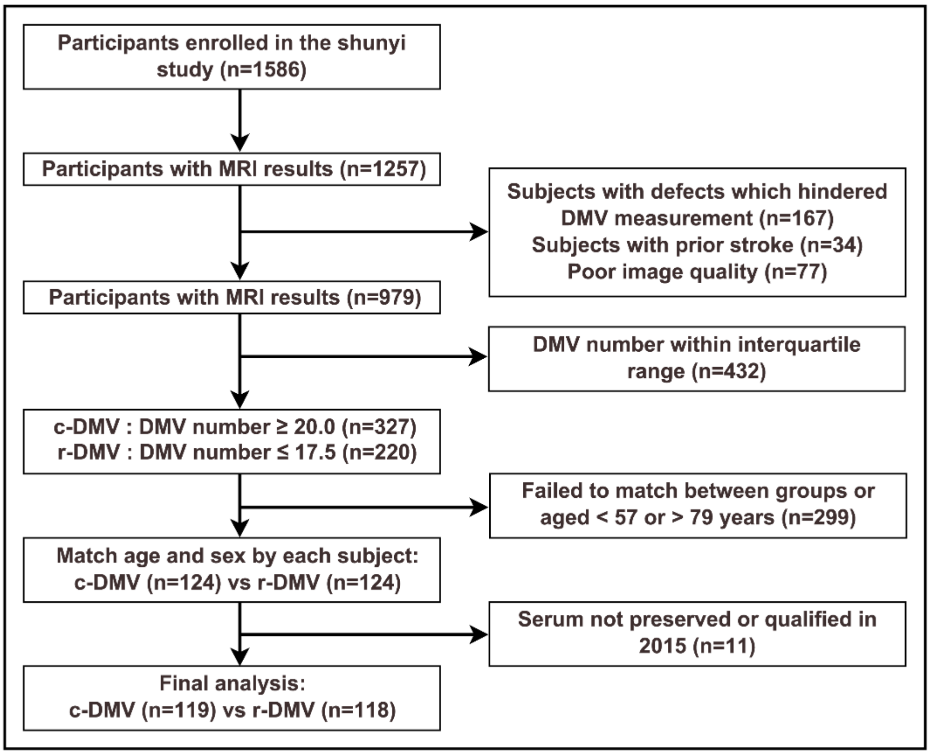

Figure S5. Flowchart of information about the enrolled participants

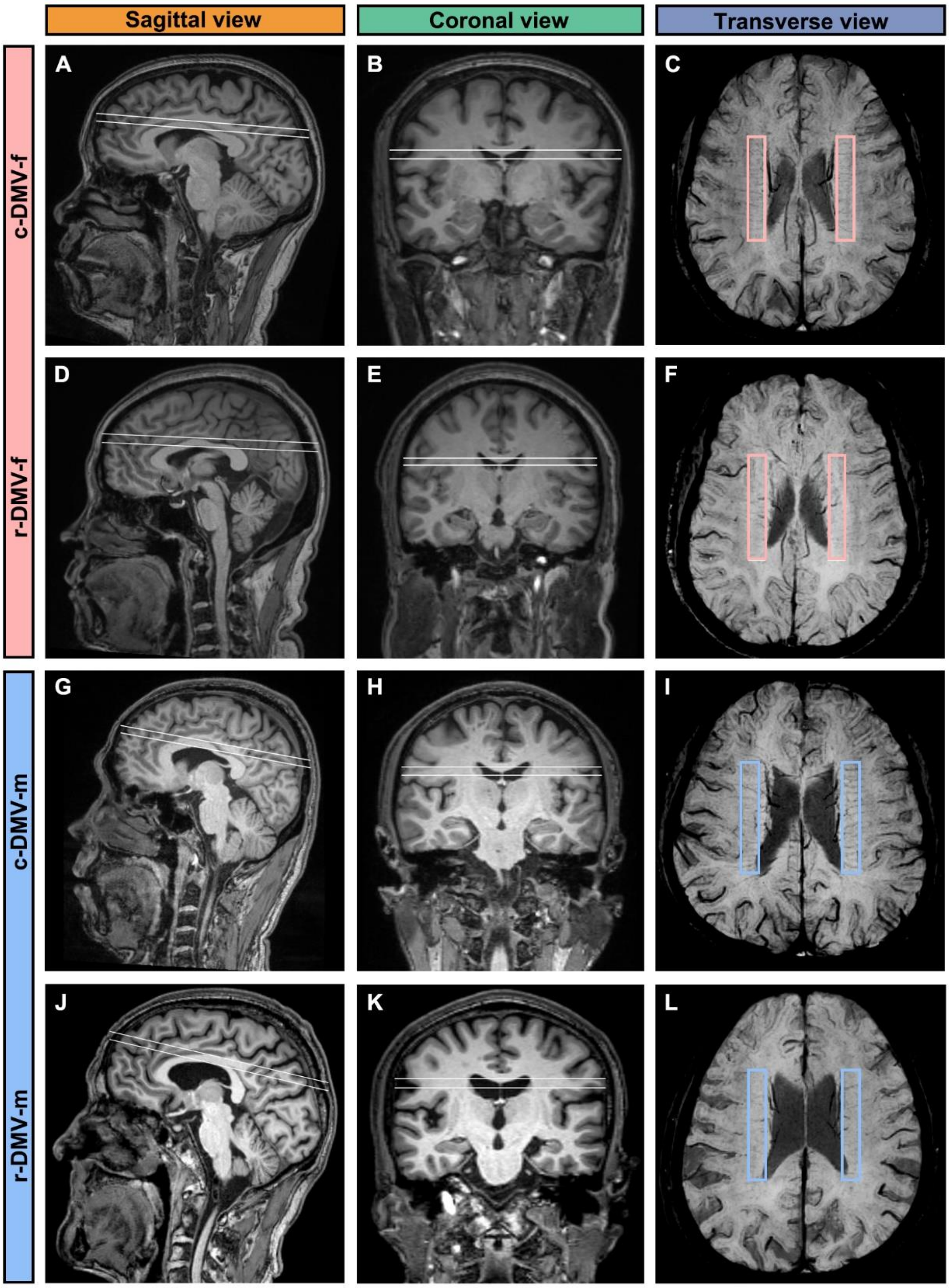

Figure S6. Images used for DMV assessment

(A-C) CTRL74, a female with preserved DMVs. (D-F) DMV105, a female with reduced DMVs. (G-I) CTRL20, a male with preserved DMVs. (J-L) DMV15, a male with reduced DMVs. (A, D, G, and J) Sagittal view of the selected block. (B, E, H, and K) Coronal view of the selected block. (C, F, I, and L) Minimum intensity projection of the selected block in 3T SWI.

Table S1. Detailed information of 237 participants

Control group (n=119)

ID	DMV	Age	BMI	HTN	DM	Hyperlipidemia	MMSE score	MOCA score	BPF	GMF	WMF	SBI	Lacune	CMB	WMH volume	logWMH	HGB	hsCRP
									**Male**									
CTRL1	21	76	24.23	-	-	-	29	24	74.55	40.90	33.65	-	-	-	5114.36	3.71	181	0.62
CTRL2	20	75	21.80	-	-	+	26	14	73.33	38.03	35.30	+	+	-	22478.37	4.35	128	1.15
CTRL3	20	75	22.53	+	-	+	19	8	73.19	37.49	35.70	+	+	-	8579.72	3.93	138	8.19
CTRL4	20	74	25.76	+	-	-	27	16	75.19	39.85	35.35	-	-	-	9233.74	3.97	154	2.76
CTRL5	20	74	29.28	-	-	+	25	20	72.47	40.33	32.14	-	-	-	1971.35	3.29	154	4.23
CTRL6	20	74	30.38	+	-	-	29	21	77.30	40.69	36.60	-	-	-	3084.11	3.49	158	1.34
CTRL7	21	73	23.95	-	-	-	29	18	74.08	39.05	35.03	-	-	-	12271.35	4.09	157	0.7
CTRL8	21	72	26.06	-	-	+	27	28	73.76	38.47	35.29	-	-	-	4001.60	3.60	150	0.6
CTRL9	20	72	21.19	-	-	-	29	20	73.24	41.86	31.38	-	-	-	1552.90	3.19	146	0.68
CTRL10	21.5	71	21.83	+	-	+	27	22	78.08	40.09	37.98	-	-	-	4482.04	3.65	159	0.46
CTRL11	21	70	20.61	-	-	-	27	19	72.84	40.23	32.62	-	-	-	9478.61	3.98	149	0.22
CTRL12	20.5	70	33.90	+	+	+	27	17	75.63	40.45	35.18	+	+	+	9676.98	3.99	163	10.53
CTRL13	20	69	32.72	+	-	-	28	18	75.69	41.29	34.40	+	+	-	7327.48	3.86	154	0.67
CTRL14	20	69	35.63	+	-	-	28	21	77.64	40.63	37.00	-	-	-	12035.78	4.08	173	0.36
CTRL15	20.5	68	22.49	-	+	-	27	22	70.47	36.97	33.50	+	+	-	18851.82	4.28	143	21.91
CTRL16	20	68	28.83	+	-	+	N/A	N/A	77.25	41.22	36.03	-	-	+	2067.44	3.32	152	2.25
CTRL17	21	67	27.16	+	+	-	29	22	74.51	40.12	34.40	-	-	-	9261.63	3.97	137	3.19
CTRL18	20.5	67	21.41	-	-	-	27	16	75.62	41.35	34.27	-	-	+	2619.17	3.42	171	10.21
CTRL19	21	68	19.76	-	-	-	25	23	76.47	42.59	33.87	-	-	-	4795.10	3.68	147	0.97
CTRL20	21	66	23.04	-	-	-	30	21	76.96	42.45	34.52	-	-	-	3254.59	3.51	167	0.69
CTRL21	20.5	66	28.01	-	+	-	25	21	78.06	43.94	34.11	-	-	-	2507.58	3.40	174	0.53
CTRL22	20	65	25.72	-	-	+	26	23	75.25	39.79	35.46	-	-	-	3294.88	3.52	167	0.66
CTRL23	21	65	23.65	+	-	-	26	15	76.93	42.01	34.92	-	-	-	7181.79	3.86	147	1.29
CTRL24	21.5	64	23.87	+	-	-	27	24	78.29	41.75	36.54	-	-	-	2358.80	3.37	156	1.02
CTRL25	20	64	27.55	-	-	-	29	23	74.69	39.89	34.80	-	-	+	4224.77	3.63	144	0.18
CTRL26	21	64	26.25	-	-	-	25	15	77.89	40.78	37.11	-	-	-	3741.23	3.57	160	0.64
CTRL27	20.5	64	27.31	+	-	+	30	25	76.65	42.27	34.38	+	-	-	4587.42	3.66	162	0.59
CTRL28	20.5	63	33.15	-	-	+	27	20	77.95	41.09	36.86	-	-	-	5108.16	3.71	173	5.21
CTRL29	20.5	62	28.43	+	-	-	27	20	78.46	41.13	37.33	-	-	-	4900.48	3.69	165	1.26

CTRL30	20	62	26.09	+	-	+	27	16	77.84	40.96	36.88	-	-	-	1664.49	3.22	169	0.91
CTRL31	20	61	20.20	-	-	-	24	15	76.82	40.73	36.09	-	-	-	2795.85	3.45	169	0.3
CTRL32	20	61	26.72	+	-	+	28	22	79.17	43.42	35.75	-	-	+	5765.27	3.76	152	1.8
CTRL33	20	61	28.17	-	-	-	27	19	75.90	42.55	33.36	-	-	-	1983.75	3.30	167	0.79
CTRL34	20	61	27.87	+	+	+	27	28	79.59	42.79	36.80	-	-	-	3496.36	3.54	163	3.5
CTRL35	22	61	24.84	+	-	+	21	13	78.30	41.59	36.72	-	-	-	1834.97	3.26	138	0.74
CTRL36	20	60	23.39	-	-	-	28	27	78.64	42.80	35.84	-	-	-	1456.82	3.16	147	0.63
CTRL37	20	59	31.44	+	-	-	29	18	76.35	40.53	35.82	-	-	-	2665.66	3.43	155	18.96
CTRL38	20	59	22.66	-	-	-	29	20	81.82	42.21	39.62	-	-	-	2926.03	3.47	142	0.49
CTRL39	22	59	N/A	-	-	-	30	19	76.20	40.48	35.72	+	+	-	8217.06	3.91	168	N/A
CTRL40	20.5	58	36.94	+	+	+	14	9	77.79	44.11	33.68	-	-	-	4699.01	3.67	171	5.41
CTRL41	20.5	58	24.61	-	+	-	29	21	77.73	43.43	34.30	-	-	-	2938.43	3.47	152	0.42
CTRL42	21	58	24.06	-	-	-	26	18	79.05	42.09	36.96	-	-	-	1726.48	3.24	165	1.09
CTRL43	20	57	21.64	-	-	+	26	14	78.69	43.21	35.48	-	-	-	3914.81	3.59	155	0.17
CTRL44	20	57	32.29	+	-	-	28	25	82.54	44.00	38.54	-	-	-	1962.05	3.29	162	0.47
**Female**																		
CTRL45	21.5	79	25.09	-	+	+	25	19	71.89	38.27	33.61	-	-	-	4234.07	3.63	134	1.61
CTRL46	21.5	78	24.94	-	-	+	22	17	76.29	42.04	34.25	-	-	+	5170.15	3.71	123	9.99
CTRL47	20.5	76	29.08	+	+	+	17	8	73.00	41.22	31.78	+	-	-	4875.69	3.69	136	2.53
CTRL48	20	75	27.07	+	+	+	25	17	69.71	39.26	30.45	+	+	-	13018.36	4.11	110	8.82
CTRL49	22	74	21.46	-	-	-	23	13	75.00	43.65	31.35	-	-	-	3539.75	3.55	120	0.8
CTRL50	20	74	22.17	+	-	+	29	21	75.57	41.28	34.29	-	-	-	2938.43	3.47	137	0.89
CTRL51	22	74	26.41	+	+	+	22	17	77.13	44.03	33.10	-	-	-	2609.87	3.42	149	4.76
CTRL52	21	73	25.04	+	-	+	23	23	74.03	38.98	35.05	-	-	-	3074.81	3.49	146	1.06
CTRL53	22	73	31.80	+	+	+	24	12	74.76	41.39	33.37	+	+	-	4243.37	3.63	148	1.04
CTRL54	22.5	72	23.08	-	-	+	29	18	75.53	39.26	36.26	-	-	-	7479.36	3.87	149	0.16
CTRL55	20.5	72	27.18	+	-	+	N/A	N/A	75.50	40.59	34.91	+	+	-	13229.13	4.12	141	0.56
CTRL56	21	72	33.86	+	-	-	16	6	78.68	43.51	35.17	-	-	-	8908.28	3.95	148	2.5
CTRL57	20	72	24.78	-	-	+	26	22	70.94	40.04	30.89	+	+	-	7358.47	3.87	145	0.78
CTRL58	21	72	26.06	+	-	-	26	18	76.07	41.22	34.84	-	-	+	3214.29	3.51	138	0.42
CTRL59	20	71	28.73	-	-	+	9	7	75.28	40.63	34.65	-	-	-	15157.09	4.18	135	1.37
CTRL60	21.5	71	26.94	-	-	+	26	19	74.55	40.93	33.62	-	-	-	2867.14	3.46	137	3.06
CTRL61	21	71	25.11	+	-	-	28	20	74.17	42.54	31.63	-	-	-	3772.22	3.58	133	4.52

CTRL62	20	70	29.40	-	-	+	17	5	74.38	41.29	33.08	-	-	-	1776.08	3.25	131	5.1
CTRL63	22.5	70	22.62	-	-	-	26	17	73.10	41.57	31.53	-	-	-	2423.89	3.38	162	0.14
CTRL64	21	73	26.06	+	-	-	26	18	76.07	41.22	34.84	-	-	+	3214.29	3.51	138	0.42
CTRL65	21.5	69	28.94	+	+	+	23	12	76.49	39.89	36.60	-	-	-	2191.42	3.34	144	5.52
CTRL66	20.5	69	25.78	+	-	+	29	18	76.64	42.26	34.39	-	-	-	2262.71	3.35	148	2.41
CTRL67	21	69	23.08	+	+	+	22	13	78.76	43.18	35.58	-	-	+	18978.91	4.28	139	0.89
CTRL68	21	69	24.57	-	-	+	26	15	74.72	42.06	32.66	-	-	-	13350.02	4.13	120	1.42
CTRL69	21.5	68	32.96	+	-	-	24	16	80.21	42.33	37.88	-	-	+	1081.76	3.03	127	2.46
CTRL70	21.5	68	22.87	+	-	-	23	16	72.27	40.45	31.82	+	+	+	6617.67	3.82	143	1.18
CTRL71	20.5	68	26.92	-	-	-	28	19	75.30	41.01	34.29	-	-	-	11846.71	4.07	124	2.18
CTRL72	20.5	68	23.56	-	-	-	18	11	76.33	43.74	32.59	-	-	-	4863.29	3.69	147	2.38
CTRL73	21.5	67	N/A	+	-	-	25	18	79.12	42.84	36.28	-	-	-	11146.20	4.05	136	2.09
CTRL74	22	67	26.13	-	+	-	28	21	74.39	39.52	34.87	-	-	-	2467.29	3.39	129	0.68
CTRL75	20.5	67	27.13	-	-	-	23	15	78.27	44.65	33.62	-	-	-	2740.05	3.44	137	2.49
CTRL76	20.5	67	25.29	+	-	+	23	10	74.41	41.90	32.52	-	-	-	15451.55	4.19	129	0.75
CTRL77	20	67	23.74	-	-	-	30	26	77.52	40.12	37.40	-	-	-	20330.34	4.31	149	0.26
CTRL78	20	67	25.47	+	-	+	23	18	77.92	41.63	36.29	-	-	-	1949.65	3.29	136	1.85
CTRL79	20.5	66	29.32	+	-	+	28	21	75.08	40.35	34.73	-	-	-	3490.16	3.54	142	0.89
CTRL80	22.5	66	21.11	-	-	+	26	14	80.07	43.42	36.65	-	-	-	1571.50	3.20	142	0.78
CTRL81	22.5	66	27.82	+	-	-	22	13	75.69	42.70	32.99	-	-	-	1329.73	3.12	124	0.54
CTRL82	22	66	23.39	-	+	+	20	12	74.95	43.28	31.67	-	-	+	8685.11	3.94	136	1.18
CTRL83	21.5	66	33.83	+	-	-	24	15	77.83	39.74	38.09	-	-	-	7067.11	3.85	130	0.29
CTRL84	21.5	65	31.40	+	-	-	29	26	77.64	43.07	34.57	-	-	-	2944.63	3.47	145	1.47
CTRL85	21.5	65	30.12	-	-	-	23	13	78.15	40.82	37.33	-	-	-	889.59	2.95	142	1.12
CTRL86	20.5	65	N/A	-	-	-	27	19	75.85	41.35	34.50	-	-	-	2752.45	3.44	128	N/A
CTRL87	23	64	25.95	-	-	-	15	12	73.91	40.27	33.64	-	-	-	7404.97	3.87	177	0.43
CTRL88	21.5	64	20.99	-	-	+	26	20	78.59	44.88	33.71	-	-	-	2203.82	3.34	133	0.47
CTRL89	21	64	33.24	-	-	+	28	14	77.15	43.15	34.00	-	-	+	3347.58	3.52	143	1.24
CTRL90	20.5	64	22.89	-	-	+	29	18	76.36	41.43	34.93	-	-	-	5514.21	3.74	127	0.77
CTRL91	21	64	30.96	+	+	-	23	14	80.34	46.39	33.94	-	-	-	5356.13	3.73	149	2.05
CTRL92	22	63	33.78	+	-	-	30	24	75.33	42.22	33.11	+	+	+	4990.37	3.70	144	3.6
CTRL93	21.5	63	27.06	-	-	+	30	25	73.89	39.02	34.87	-	-	-	2272.01	3.36	129	0.54
CTRL94	21.5	62	27.32	-	-	+	23	15	74.80	40.96	33.84	-	-	-	3074.81	3.49	135	2.91

CTRL95	21	62	22.78	+	+	+	28	17	79.26	44.12	35.14	-	-	-	1648.99	3.22	143	1.51
CTRL96	21	63	28.47	-	-	+	22	17	77.63	44.19	33.43	-	-	-	1828.77	3.26	144	1.17
CTRL97	20	62	23.78	+	-	-	26	20	78.40	44.63	33.77	+	-	-	1980.65	3.30	151	0.23
CTRL98	21	62	20.00	-	-	+	30	20	77.36	41.79	35.56	-	-	-	4940.78	3.69	135	0.54
CTRL99	21	62	21.08	-	-	-	26	16	75.98	43.11	32.87	-	-	-	2417.70	3.38	142	0.27
CTRL100	20	61	24.36	-	-	+	29	26	76.77	40.73	36.04	-	-	+	2082.94	3.32	148	2.38
CTRL101	21.5	61	31.68	+	-	-	29	23	77.53	43.86	33.68	-	-	-	3375.47	3.53	139	0.51
CTRL102	20	61	29.89	+	-	-	22	20	79.15	42.74	36.41	-	-	-	3502.56	3.54	154	2.68
CTRL103	20	61	27.11	-	-	+	30	27	79.65	42.66	36.99	-	-	-	1738.88	3.24	134	12.46
CTRL104	20.5	61	23.81	-	-	+	24	11	82.94	47.66	35.28	-	-	-	5222.84	3.72	126	1.25
CTRL105	21	60	26.04	+	-	-	29	21	78.88	43.17	35.72	-	-	+	3273.19	3.51	132	1.69
CTRL106	21.5	60	30.11	+	-	+	28	19	78.19	44.67	33.52	-	-	-	1652.09	3.22	132	1.05
CTRL107	21	59	29.08	-	+	+	26	18	78.14	42.46	35.68	-	-	-	1112.76	3.05	147	0.93
CTRL108	20.5	59	22.88	-	-	-	24	13	77.82	42.91	34.91	-	-	-	1683.09	3.23	139	0.88
CTRL109	20	59	23.80	+	+	-	26	18	77.85	41.22	36.63	-	-	-	2098.44	3.32	155	7.31
CTRL110	20	59	23.55	-	-	-	29	23	79.23	43.28	35.95	-	-	-	1965.15	3.29	128	N/A
CTRL111	20	59	24.61	+	-	+	29	20	78.98	42.82	36.16	-	-	-	1633.49	3.21	134	3.24
CTRL112	20	59	N/A	+	-	+	27	18	80.69	45.24	35.45	-	-	-	1720.28	3.24	148	0.76
CTRL113	20	59	28.54	-	-	-	26	25	78.71	43.56	35.15	-	-	-	1177.85	3.07	104	2.01
CTRL114	20.5	58	27.89	-	-	+	30	24	77.69	43.13	34.56	-	-	-	1986.85	3.30	154	3.12
CTRL115	21	58	29.68	-	-	+	29	23	78.39	41.99	36.40	-	-	-	1878.36	3.27	147	0.93
CTRL116	21	58	28.00	-	-	-	30	18	79.58	43.80	35.79	-	-	-	10848.63	4.04	146	1.24
CTRL117	21.5	57	33.28	+	-	-	27	21	80.64	41.88	38.77	-	-	-	2888.84	3.46	124	1.85
CTRL118	20.5	57	29.25	+	-	+	29	25	77.47	41.97	35.51	-	-	-	3635.84	3.56	158	1.77
CTRL119	21	57	19.90	-	-	-	26	15	81.08	44.34	36.74	-	-	-	1574.60	3.20	148	0.53

*N/A: not available. DMV=deep medullary vein number, BMI=body mass index, HTN=hypertension, DM=diabetes, MMSE=mini mental state examination, MOCA=Montreal cognitive assessment, BPF=brain parenchyma fraction, GMF=gray matter fraction, WMF=white matter fraction, SBI=silent brain infarction, CMB=cerebral microbleeds, WMH=white matter hyperintensity, HGB=hemoglobin, hsCRP=hypersensitive C-reactive protein.

DMV reduction group (n=118)

ID	DMV	Age	BMI	HTN	DM	Hyperlipidemia	MMSE score	MOCA score	BPF	GMF	WMF	SBI	Lacune	CMB	WMH volume	logWMH	HGB	hsCRP
									**Male**									
DMV1	15	76	23.91	-	-	-	26	14	66.47	36.69	29.77	-	-	-	3800.12	3.58	149	0.92
DMV2	17.5	76	21.11	-	-	-	23	19	73.61	40.48	33.13	+	+	-	2637.77	3.42	142	3.35
DMV3	17.5	74	23.18	+	-	-	27	17	74.25	38.01	36.24	+	+	-	6868.73	3.84	137	0.61
DMV4	15	73	26.31	+	+	-	27	16	71.35	39.90	31.46	+	+	-	6003.94	3.78	142	4.51
DMV5	17	72	27.62	+	-	-	25	19	74.99	39.87	35.12	-	-	-	6952.42	3.84	173	0.35
DMV6	17	72	27.66	+	-	+	28	20	73.34	40.20	33.14	-	-	-	3716.43	3.57	161	3.43
DMV7	14	72	N/A	+	-	-	27	17	72.07	40.03	32.04	+	+	-	8728.50	3.94	157	0.55
DMV8	17	71	27.57	+	-	-	29	21	80.05	42.17	37.88	+	+	-	4237.17	3.63	163	2.45
DMV9	17	71	26.58	+	-	-	27	15	73.59	38.87	34.72	+	+	+	26396.27	4.42	158	30.96
DMV10	16.5	71	20.64	-	-	-	29	21	75.51	39.92	35.59	-	-	+	1329.73	3.12	139	1.3
DMV11	17.5	71	30.91	+	-	+	30	20	76.34	39.97	36.37	-	-	-	5486.31	3.74	163	0.69
DMV12	17.5	70	26.73	+	-	-	27	19	76.33	41.99	34.35	-	-	-	2194.52	3.34	152	1.13
DMV13	17	70	N/A	-	-	-	26	24	77.22	40.95	36.27	-	-	-	3592.45	3.56	141	N/A
DMV14	17.5	68	22.84	+	-	+	28	24	77.36	41.59	35.78	-	-	-	2024.04	3.31	155	0.39
DMV15	17.5	67	26.13	+	-	+	30	18	77.41	40.09	37.32	-	-	-	3211.20	3.51	142	0.83
DMV16	16.5	67	24.69	+	-	-	29	22	74.28	39.22	35.05	-	-	-	6602.17	3.82	152	0.53
DMV17	17	67	31.45	-	-	-	29	27	79.67	41.10	38.57	-	-	-	6264.31	3.80	168	5.78
DMV18	16.5	67	27.46	+	+	+	24	15	77.45	39.91	37.54	-	-	-	3925.78	3.59	145	0.47
DMV19	13.5	67	22.02	-	-	-	27	18	73.26	38.91	34.35	-	-	-	3564.55	3.55	153	0.45
DMV20	17.5	66	N/A	+	-	-	29	22	77.41	42.54	34.88	-	-	-	13297.32	4.12	181	1.23
DMV21	17	65	25.45	-	+	+	29	22	69.39	38.26	31.13	-	-	-	6825.34	3.83	168	4.16
DMV22	17.5	65	27.99	+	+	+	25	18	72.57	38.89	33.68	-	-	-	10662.66	4.03	156	4.43
DMV23	15.5	64	18.01	-	-	-	19	14	75.58	42.67	32.91	-	-	-	3942.70	3.60	171	N/A
DMV24	17.5	64	33.20	+	-	+	29	24	75.42	41.68	33.73	+	+	+	6688.96	3.83	151	17.03
DMV25	15.5	64	32.71	+	-	+	26	23	75.99	40.43	35.57	-	-	-	3164.70	3.50	154	1.83
DMV26	17.5	64	27.07	+	+	+	28	20	71.66	39.17	32.49	-	-	-	3632.74	3.56	180	3.54
DMV27	16.5	62	21.96	-	-	-	24	15	79.85	43.33	36.52	-	-	-	1066.27	3.03	115	1.74
DMV28	16.5	61	23.49	-	-	-	26	19	71.96	38.78	33.18	-	-	-	3186.40	3.50	141	0.15
DMV29	17.5	61	31.61	-	-	-	29	22	75.96	41.32	34.64	-	-	-	3195.70	3.50	168	5.13
DMV30	17	61	31.84	+	-	-	26	14	80.39	46.71	33.68	-	-	-	2498.29	3.40	155	2.52

DMV31	16	61	24.46	-	+	-	24	12	80.27	43.47	36.79	-	-	-	1140.66	3.06	173	1.4
DMV32	16	60	31.70	+	-	-	29	23	78.19	40.88	37.31	-	-	-	1878.36	3.27	158	1.28
DMV33	13	60	24.57	-	-	+	28	18	74.50	38.64	35.86	-	-	-	1940.36	3.29	152	2.31
DMV34	16.5	60	24.31	-	-	-	29	25	75.23	40.31	34.92	-	-	-	1546.71	3.19	165	0.93
DMV35	17	60	N/A	-	-	-	29	24	78.85	44.25	34.60	-	-	-	2299.91	3.36	172	0.24
DMV36	15	59	27.74	-	-	-	24	21	79.65	41.31	38.34	-	-	-	750.11	2.88	156	13.09
DMV37	17	59	30.66	+	-	+	28	26	78.19	43.64	34.55	-	-	-	1249.14	3.10	153	0.99
DMV38	16.5	58	29.83	-	-	-	30	26	77.67	41.57	36.09	-	-	-	2411.50	3.38	168	3.24
DMV39	16.5	58	27.13	-	+	+	26	23	78.35	42.13	36.21	-	-	-	3586.25	3.55	152	8.1
DMV40	16.5	57	26.86	-	-	+	30	28	78.95	42.10	36.86	-	-	-	3012.82	3.48	151	1.55
DMV41	17.5	57	21.30	+	-	-	23	17	81.32	45.71	35.61	-	-	-	1813.27	3.26	183	0.65
DMV42	17	57	28.68	+	-	+	29	23	75.43	41.34	34.09	+	+	-	2709.06	3.43	147	1.86
DMV43	17	57	24.02	+	-	-	29	16	73.90	39.65	34.26	+	+	+	15752.21	4.20	161	N/A
								**Female**										
DMV44	15	78	23.08	+	+	+	30	19	70.33	39.28	31.05	-	-	-	2743.15	3.44	130	1.13
DMV45	15	78	22.86	-	-	+	28	17	72.79	37.87	34.91	-	-	-	2287.51	3.36	125	1.34
DMV46	15.5	76	30.71	+	-	+	26	16	74.70	40.09	34.61	+	+	-	2281.31	3.36	142	0.62
DMV47	16	76	22.64	+	+	-	18	13	71.69	39.26	32.43	-	-	-	2098.44	3.32	125	0.37
DMV48	17	76	27.06	+	-	+	14	12	72.34	41.72	30.62	+	+	-	3942.70	3.60	150	1.19
DMV49	16.5	75	27.57	+	-	-	29	16	75.72	40.60	35.12	-	-	+	2702.86	3.43	144	3.42
DMV50	16	74	31.50	+	-	-	27	17	71.97	40.88	31.09	+	+	+	53421.77	4.73	131	1.49
DMV51	17	74	26.66	+	-	+	22	15	74.23	40.86	33.37	-	-	+	6109.33	3.79	139	0.87
DMV52	16.5	74	27.82	+	+	+	14	8	70.84	37.88	32.96	-	-	-	6065.94	3.78	133	8.47
DMV53	17.5	73	26.80	+	-	+	25	12	73.38	39.56	33.81	+	+	-	12299.25	4.09	135	2.07
DMV54	17	72	23.69	-	-	+	26	16	77.56	40.53	37.03	-	-	-	3862.11	3.59	138	1.89
DMV55	17.5	71	22.35	+	-	-	N/A	N/A	75.44	42.75	32.69	-	-	+	9267.83	3.97	132	0.97
DMV56	17	71	26.62	+	+	+	23	12	75.38	42.13	33.25	+	+	-	13663.08	4.14	142	0.29
DMV57	17	71	30.96	+	-	+	28	20	77.01	41.67	35.34	-	-	-	6096.93	3.79	138	4.14
DMV58	17	71	27.74	+	+	+	25	18	72.87	39.93	32.93	-	-	-	13015.26	4.11	137	0.53
DMV59	16.5	70	21.60	+	-	-	26	19	75.66	44.80	30.86	-	-	-	16282.25	4.21	146	0.24
DMV60	16	70	26.84	-	+	+	27	18	70.79	38.70	32.09	-	-	-	2873.34	3.46	143	3.35
DMV61	16.5	70	N/A	+	-	-	30	23	71.65	39.10	32.54	+	+	-	4531.63	3.66	135	2.28
DMV62	17	70	N/A	-	-	-	29	27	74.96	42.70	32.26	-	-	-	12193.86	4.09	115	N/A

DMV63	16.5	69	25.42	+	-	+	N/A	N/A	72.70	37.29	35.41	-	-	-	4373.55	3.64	133	3.18
DMV64	14.5	69	26.41	+	-	+	25	17	72.49	41.89	30.60	+	+	-	5579.30	3.75	139	6.78
DMV65	16	69	34.53	+	-	+	N/A	N/A	82.31	44.53	37.78	-	-	-	1989.95	3.30	150	3.51
DMV66	17	69	29.48	+	-	-	26	23	75.84	41.56	34.28	+	+	+	4528.53	3.66	154	0.27
DMV67	17.5	69	23.60	-	-	-	27	16	73.23	40.72	32.51	-	-	-	2941.53	3.47	142	2.74
DMV68	17.5	69	27.04	+	-	-	15	N/A	75.71	40.66	35.05	-	-	-	38859.80	4.59	132	0.26
DMV69	14.5	68	26.42	+	-	+	24	17	74.10	42.87	31.22	-	-	-	1038.37	3.02	148	27.47
DMV70	16	68	21.94	+	-	+	30	21	75.09	42.12	32.97	-	-	-	6800.54	3.83	132	0.68
DMV71	17	68	29.28	-	-	-	21	9	72.48	39.84	32.64	-	-	-	3753.63	3.57	138	1.44
DMV72	16.5	68	24.76	-	-	-	30	16	74.00	39.70	34.30	-	-	-	4649.41	3.67	143	3.78
DMV73	16.5	68	25.04	+	-	+	25	18	77.45	43.64	33.82	-	-	-	2430.09	3.39	131	0.47
DMV74	15.5	67	20.46	+	+	+	24	12	76.35	41.65	34.71	-	-	-	6047.34	3.78	143	1.21
DMV75	17.5	67	29.02	+	-	-	N/A	N/A	77.14	42.38	34.76	-	-	-	3905.51	3.59	129	N/A
DMV76	13	67	24.86	+	+	-	17	6	74.94	39.25	35.68	-	-	-	5557.60	3.74	141	0.61
DMV77	16.5	67	30.45	+	-	+	26	15	75.79	39.87	35.91	-	-	-	1822.57	3.26	125	11.5
DMV78	15	67	35.97	+	-	-	26	16	76.51	42.75	33.76	-	-	-	34092.60	4.53	143	1.26
DMV79	17.5	66	32.83	-	-	+	27	16	77.10	44.21	32.89	-	-	-	5055.46	3.70	137	2.16
DMV80	17	66	21.37	+	-	+	22	11	74.44	40.81	33.63	-	-	-	6112.43	3.79	131	0.71
DMV81	16.5	66	26.49	+	+	+	27	11	74.96	43.42	31.54	+	+	-	4243.37	3.63	146	1.87
DMV82	16.5	66	20.89	-	-	+	29	17	75.02	41.73	33.29	-	-	-	4714.51	3.67	139	0.51
DMV83	13	65	23.19	+	+	+	17	10	67.41	37.82	29.59	+	+	+	26535.76	4.42	145	0.39
DMV84	15.5	65	30.49	+	-	+	17	5	73.83	37.69	36.14	-	-	-	3961.30	3.60	162	5.76
DMV85	16	65	26.01	+	+	+	25	24	74.95	41.19	33.76	-	-	-	3068.61	3.49	148	1.42
DMV86	17.5	64	24.27	-	+	+	24	17	79.41	42.63	36.78	+	+	-	8917.58	3.95	139	0.63
DMV87	17.5	64	26.64	+	-	+	29	20	77.01	43.67	33.33	-	-	-	1267.74	3.10	134	3.06
DMV88	17	64	22.23	-	+	+	29	18	75.47	41.74	33.73	-	-	-	3803.22	3.58	149	0.59
DMV89	16	64	24.27	-	-	-	30	23	77.85	43.61	34.24	-	-	-	4559.53	3.66	150	2.45
DMV90	16	63	32.16	-	-	-	25	18	80.30	43.25	37.05	-	-	-	1131.36	3.05	122	4.31
DMV91	16.5	63	26.03	-	-	+	20	10	80.23	44.01	36.23	-	-	-	1348.33	3.13	138	1.09
DMV92	17	63	21.72	+	-	-	27	17	75.21	40.64	34.57	-	-	-	2070.54	3.32	138	1.42
DMV93	16.5	63	24.27	+	-	-	24	19	77.97	43.25	34.72	-	-	-	5802.47	3.76	138	8.1
DMV94	15.5	63	26.60	+	-	-	28	23	77.65	40.21	37.44	-	-	-	4454.14	3.65	151	0.26
DMV95	17.5	62	25.72	+	+	+	30	24	77.15	43.20	33.95	-	-	-	706.71	2.85	118	3.49

DMV96	16	62	22.70	-	-	-	26	18	76.72	41.22	35.50	-	-	-	2473.49	3.39	134	0.5
DMV97	17	62	27.47	+	-	+	29	19	78.75	42.68	36.08	-	-	-	4106.98	3.61	136	2.74
DMV98	17	61	32.00	+	-	+	30	17	75.61	39.33	36.28	+	+	-	8638.61	3.94	149	0.75
DMV99	16	61	30.24	+	+	+	29	22	76.24	42.96	33.28	+	+	+	3905.51	3.59	128	1.49
DMV100	16	61	31.46	-	-	+	27	15	78.93	43.07	35.86	-	-	-	2457.99	3.39	122	2.71
DMV101	15.5	61	24.37	-	-	-	28	23	78.46	42.04	36.43	-	-	-	2194.52	3.34	149	0.24
DMV102	17.5	60	28.35	-	-	+	30	24	77.97	42.05	35.92	-	-	-	561.03	2.75	154	1.37
DMV103	17.5	60	21.61	-	-	+	29	22	77.35	42.87	34.47	-	-	-	1397.92	3.15	134	0.37
DMV104	17.5	60	26.19	-	-	-	23	16	83.23	44.44	38.79	-	-	-	2526.18	3.40	128	N/A
DMV105	14.5	59	31.97	-	+	+	30	25	81.68	43.49	38.19	-	-	-	1388.63	3.14	141	0.84
DMV106	16	59	24.76	-	+	+	26	13	75.39	43.37	32.02	-	-	-	2117.03	3.33	138	3.2
DMV107	17.5	59	23.83	-	-	-	27	18	77.61	40.64	36.97	-	-	-	1590.10	3.20	127	1.41
DMV108	16.5	59	29.73	-	-	+	29	26	78.67	41.60	37.08	-	-	-	1844.27	3.27	159	0.63
DMV109	17.5	58	29.49	+	-	+	26	20	73.62	42.52	31.10	-	-	-	1834.97	3.26	117	1.11
DMV110	17	58	30.12	-	-	+	26	13	79.94	45.99	33.94	-	-	-	1153.05	3.06	139	2.37
DMV111	17.5	58	31.59	+	-	+	28	18	78.04	42.03	36.01	-	-	-	1205.75	3.08	137	1.9
DMV112	16.5	58	31.56	+	-	+	30	22	77.46	42.30	35.16	-	-	-	2569.58	3.41	132	2.76
DMV113	17.5	58	26.53	-	-	-	26	19	77.84	43.81	34.03	-	-	-	1428.92	3.16	142	2.81
DMV114	17.5	58	29.09	+	-	-	26	25	76.96	42.57	34.39	-	-	-	1906.26	3.28	129	0.95
DMV115	14.5	58	30.18	-	-	+	30	24	76.17	40.17	36.00	-	-	-	1797.77	3.25	140	1.5
DMV116	15.5	57	27.03	-	+	+	30	27	76.91	42.96	33.94	-	-	-	2008.55	3.30	130	3.98
DMV117	16.5	57	31.08	-	-	+	27	18	78.71	43.29	35.42	-	-	-	2048.84	3.31	139	1.04
DMV118	16.5	57	19.77	-	-	-	24	13	74.52	40.36	34.16	-	-	-	4894.28	3.69	121	0.96

*N/A: not available. DMV=deep medullary vein number, BMI=body mass index, HTN=hypertension, DM=diabetes, MMSE=mini mental state examination, MOCA=Montreal cognitive assessment, BPF=brain parenchyma fraction, GMF=gray matter fraction, WMF=white matter fraction, SBI=silent brain infarction, CMB=cerebral microbleeds, WMH=white matter hyperintensity, HGB=hemoglobin, hsCRP=hypersensitive C-reactive protein.

Table S5. Reagents and resources table

Reagent or resource	Source	Identifier
Adobe Photoshop	Adobe	https://www.adobe.com/products/photoshop/
Alexa Fluor™ 488 Phalloidin	Thermo Fisher Scientific	Cat# A12379
Amicon Ultra-4 Centrifugal Filter 30kD	Merck	Cat# UFC8030
Anti-ALIX [EPR15314] N-terminal antibody	Abcam	Cat# ab186429, RRID: AB_2754981
Anti-PSD 95 antibody	Synaptic Systems	Cat# 124 011, RRID: AB_10804286
Anti-Synapsin I antibody	Abcam	Cat# ab254349
B-27 Supplement (50X), minus vitamin A	GIBCO	Cat# 12587-010
Beta-actin antibody	GenTex	Cat# GTX124213
CD63 (H-193) antibody	Santa Cruz Biotechnology	Cat# Sc-15363, RRID: AB_648179
Cell Counting Kit-8	MedChem Express	Cat# HY-K0301
CFX Connect Real-Time PCR Detection System	Bio-Rad	Cat# 1855202
CFX Maestro Software 2.3	Bio-Rad	Cat# 12004110
Corning Matrigel Growth Factor Reduced (GFR) Basement Membrane Matrix	Merck	Cat# CLS354230
DAPI Solution	Solarbio	Cat# C0065
DEPC-Treated Water	Invitrogen	Cat# 750023
Dulbecco's Modified Eagle Medium (DMEM) with high glucose	Hyclone	Cat# SH30243.01
EDTA-free Protease Inhibitor Cocktail	Roche	Cat# 04693132001
Fetal Bovine Serum	GIBCO	Cat# 10099-141C
Fiji/ImageJ Software	Fiji	RRID: SCR_002285
GFAP antibody [2A5] - Astrocyte Marker	Abcam	Cat# ab4648, RRID:AB_449329
Goat anti-Mouse IgG (H+L) Highly Cross-Adsorbed Secondary Antibody, Alexa Fluor Plus 488	Thermo Fisher Scientific	Cat# A32723, RRID: AB_2633275
Goat anti-Rabbit IgG (H+L) Highly Cross-Adsorbed Secondary Antibody, Alexa Fluor 594	Thermo Fisher Scientific	Cat# A-11037, RRID: AB_2534095
GraphPad Prism	GraphPad	RRID: SCR_002798
Horseradish enzyme labeled goat anti-mouse IgG (H+L) (affinity purification) antibody	ZSGB-Bio	Cat# ZB_2305, RRID: AB_2747415

Human amyloid beta peptide 1-40, Aβ1-40 ELISA Kit	CUSABIO	Cat# CSB-E08299h
Human amyloid beta peptide 1-42, Aβ1-42 ELISA Kit	CUSABIO	Cat# CSB-E10684h
Human Complement 3,C3 ELISA Kit	CUSABIO	Cat# CSB-E08665h
Human Serum Albumin antibody [15C7]	Abcam	Cat# ab10241, RRID: AB_296978
Image Lab Software	Bio-Rad	RRID: SCR_014210
imaris	imaris	RRID： SCR_007370
Immobilon Western Chemiluminescent HRP Substrate	Merck Millipore	Cat# WBKLS0500
LAMP2 antibody	Proteintech	Cat# 27823-1-AP, RRID: AB_2880983
LAS AF Software	Leica	https://www.leica-microsystems.com/products/microscopesoftware/p/leica-las-x-ls/
L-Glutamine	Hyclone	Cat# SH30034.01
MAP2 Antibody	Cell Signaling Technology	Cat# 4542, RRID: AB_10693782
Multiskan FC Microplate Photometer	Thermo Fisher Scientific	Cat# 51119000
NanoDrop 2000/2000c Spectrophotometers	Thermo Fisher Scientific	Cat# ND2000
Neurobasal medium	Thermo Fisher Scientific	Cat# 21103049
Penicillin-Streptomycin	GIBCO	Cat# 15140-122
Peroxidase-AffiniPure Goat Anti-Rabbit IgG (H+L) antibody	Jackson ImmunoResearch Labs	Cat# 111-035-003 RRID: AB_2313567
Phospho-Tau (Ser396) Polyclonal Antibody	Thermo Fisher Scientific	Cat# 44-752G, RRID: AB_2533745
pHrodo Red, succinimidyl ester	Invitrogen	Cat# p36600
Pierce BCA Protein Assay Kit	Thermo Fisher Scientific	Cat# 23225
PrimeScript™ RT Master Mix (Perfect Real Time)	Takara	Cat# RR036A
qEVoriginal/35nm Gen 2 Column	IZON	Cat# ICO-35
Qsonica Q800R Ultrasonicator	Qsonica	Cat# 15-338-330
R	https://www.r-project.org	RRID: SCR_001906
R Studio (V4.0.5)	https://rstudio.com/	RRID: SCR_001905
RIPA	Solarbio	Cat# R0010
RPMI 1640 medium with HEPES, L-glutamine	Hyclone	Cat# SH30255.01

RPMI Medium modified without Calcium Nitrate, with L-glutamine	Hyclone	Cat# SH30809.01
Syn-PER Synaptic Protein Extraction Reagent	Thermo Fisher Scientific	Cat# 87793
Tau (phospho S396) antibody [E178]	Abcam	Cat# ab32057, RRID: AB_778254
TB Green® Premix Ex Taq™ II (Tli RNaseH Plus)	Takara	Cat# RR820A
TRIzol	Invitrogen	Cat# 15596018
Trypsin 0.25% protease with porcine trypsin, HBSS, EDTA; without calcium, magnesium	Hyclone	Cat# SH30042.01

Table S6. Sequences of the primers

Gene	Primer	Sequence (5'→3')	Organism
Acta2	Forward	AGATCAAGATTATTGCTCCTCC	Rat
	Reverse	GGAAGGTAGATAGAGAAGCCA	
Actb	Forward	AAGTCCCTCACCCTCCCAAAAG	Rat
	Reverse	AAGCAATGCTGTCACCTTCCC	
Amigo2	Forward	GTTCGCCACAACAACATCAC	Rat
	Reverse	GTTTCTGCAAGTGGGAGAGC	
Axl	Forward	GAAGCCACCTTGAACAGTC	Rat
	Reverse	CACCTTATGCCGATCTACCA	
B3gnt5	Forward	TGCTCCTGGATGAAAGGTCC	Rat
	Reverse	ACATGCTTGATCCGTGTGGT	
Clcf1	Forward	GGACCTACCTGAACTACCTG	Rat
	Reverse	CACTTCCAAGTTGACCGTG	
C3	Forward	ACAGGAGAAACTTAAGGTAAGGG	Rat
	Reverse	TAGTACCGCTTCTTGGCAG	
Cd109	Forward	GTCGCTCACAGGTACCTCAA	Rat
	Reverse	CTGTGAAGTTGAGCGTTGGC	
Cd44	Forward	CACTCAAGTGGGAATCAAGAC	Rat
	Reverse	GATAAGCCACTCTGGAATCTG	
Cldn5	Forward	TGACTGCCTTCCTGGACCACAA	Mouse
	Reverse	CATACACCTTGCACTGCATGTGC	
Cnn1	Forward	AAACAAGAGCGGAGATTTGAG	Rat
	Reverse	AAACTTATTGGTGCCCATCTG	
Cp	Forward	GATGTTTCCCCAAACGCCTG	Rat
	Reverse	GTAGCTCTGAGACGATGCTTGA	
Emp1	Forward	ACCATTGCCAACGTCTGGAT	Rat
	Reverse	TGGAACACGAAGACCACGAG	
Ereg	Forward	CATTGACCGTGATTCTCGT	Rat
	Reverse	CGATTTCTGTACCATCTGCA	
Fbln5	Forward	ATGGTTCTTTCATCTGCCG	Rat
	Reverse	TGCACTCATCCATATCACTG	
Fkbp5	Forward	GAACCCAATGCTGAGCTTATG	Rat
	Reverse	ATGTACTTGCCTCCCTTGAAG	
Gapdh	Forward	CATCACTGCCACCCAGAAGACTG	Mouse

	Reverse	ATGCCAGTGAGCTTCCCGTTCAG	
Gas6	Forward	GATATCAAGAGTGCATGAGGA	Rat
	Reverse	GTGCCCTTCTTATCACAGG	
Gbp2	Forward	GGAGATTGAAGTGGAACGG	Rat
	Reverse	TCAAACTTCTCCTGCTTCTC	
Gfap	Forward	AAATCTGTGTCAGAAGGCCA	Rat
	Reverse	CTCCTTAATGACCTCGCCA	
Ggta1	Forward	TCTCAGGATCTGGGAGTTGGA	Rat
	Reverse	GAGTTCTATGGAGCTCCCGC	
H2-T23	Forward	ATTGGAGCTGTTGTGAGGAGG	Rat
	Reverse	CCACGAGGCAACTGTCTTTTC	
Hspb1	Forward	AAATACACGCTCCCTCCAG	Rat
	Reverse	GTGATCTCCGCTGATTGTG	
Lcn2	Forward	GGAATATTCACAGCTACCCTC	Rat
	Reverse	ATACCATGGCAAACTGGTC	
Megf10	Forward	TACCGCCATGGGGAGAAAAC	Rat
	Reverse	TTATCAGCGCAGTGAGGGAC	
Mertk	Forward	CTGCTTCTGCGGGTTTGTTC	Rat
	Reverse	GGCTTTGCAAGGTAAGCTCG	
Myh11	Forward	TTCACTAAAGTAAAGCCGCTG	Rat
	Reverse	GATCTTCTGCATCTCCTCCT	
Ocln	Forward	TGGCAAGCGATCATACCCAGAG	Mouse
	Reverse	CTGCCTGAAGTCATCCACACTC	
Psmb8	Forward	CAGGAAGTTACATTGCTACCA	Rat
	Reverse	CACAACCAGACATGGTTCC	
Ptgs2	Forward	CCAACCTCTCCTACTACACC	Rat
	Reverse	CCTTATTTCCTTTCACACCCA	
Ptx3	Forward	CAATGGACTTCATCCCACC	Rat
	Reverse	GATGAACAGCTTGTCCCAC	
S100a10	Forward	ATGGAAAGGGAGTTCCCTG	Rat
	Reverse	ATAGAAAGCTCTGGAAGCCC	
S100b	Forward	AAGCACAAGCTGAAGAAGTC	Rat
	Reverse	CTGCTCTTTGATTTCCTCCA	
S1pr3	Forward	ACTGTTGAGCTTCATCGTC	Rat
	Reverse	TTGACCTTGTAGGCTATGC	

Serping1	Forward	TGGCTCAGAGGCTAACTGGC	Rat
	Reverse	GAATCTGAGAAGGCTCTATCCCCA	
Sphk1	Forward	GAACTACTATGCTGGGCAC	Rat
	Reverse	GATTCATGGGTGACAGCTG	
Spp1	Forward	TTTCTGATGAACAGTATCCCGA	Rat
	Reverse	TGATAGCCTCATCGGACTC	
Srgn	Forward	GTTCAAGGTTATCCTGCTCGGA	Rat
	Reverse	AAACAGGATCGGTCATCGGG	
Steap4	Forward	CAAACGCCGAGTACCTTGCT	Rat
	Reverse	CAGACAAACACCTGCCGACT	
Tagln	Forward	TGGCTGAAGAATGGCGTGAT	Rat
	Reverse	TCCATCGTTCTTGGTCACGG	
Tgm1	Forward	CACATAATCCTCTTCCTGAACC	Rat
	Reverse	CTCAGGATTGTTTCCGATGAG	
Timp1	Forward	TGATAGCTTCCAGTAAAGCC	Rat
	Reverse	CCCTTATAACCAGGTCCGA	
Tm4sf1	Forward	CAGAAGGACCAAAGTGTAGC	Rat
	Reverse	AATCCAGAAGGTACTGTCCC	
Vim	Forward	TCACTTCCTCTGGTTGACAC	Rat
	Reverse	ATTGATCACCTGTCCGTCTC	
ZO1	Forward	GTTGGTACGGTGCCCTGAAAGA	Mouse
	Reverse	GCTGACAGGTAGGACAGACGAT	
